# Supplementary figures and images for: Mms19 promotes spindle microtubule assembly in Drosophila neural stem cells
Source: PLoS Genet. 2020 Nov 19;16(11):e1008913. doi: 10.1371/journal.pgen.1008913 (PMC7714366; doi:10.1371/journal.pgen.1008913)

S1 Fig: *Mms19<sup>P</sup>* NBs do not display elevated levels of aneuploidy

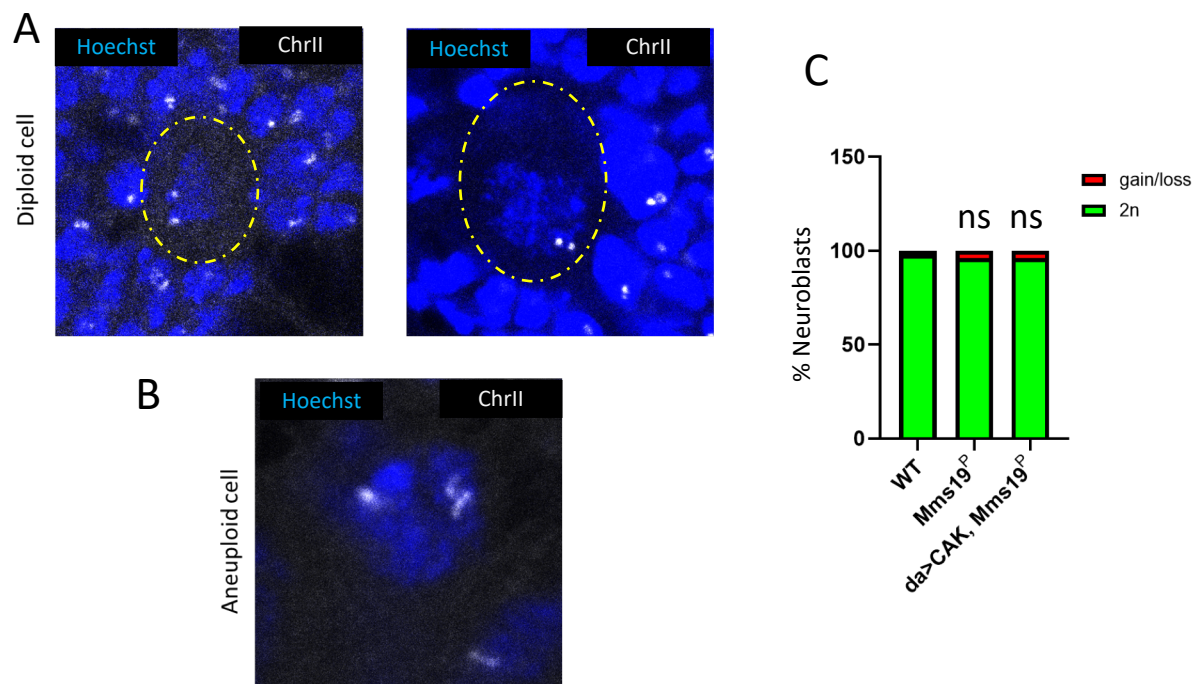

Supplement: S1 Fig — Wild type, Mms19P, and da>CAK, Mms19P brains were fixed and fluorescent in situ hybridization was performed on them. Cy5-labeled DNA probes that specifically bind to regions on the 2nd chromosome were used to determine the number of 2nd chromosomes. (A) The signal is seen as 2 dots in the WT NBs corresponding to the diploid state of the cell. (B) An example of an aneuploid Mms19P NB showing 3 dots. (C) Percentages of diploid (green) aneuploid (red) cells in each genotype were quantified. Aneuploid cells in Mms19P (and da>CAK, Mms19P) NBs were extremely rare and not significantly elevated (P = 0.1493). WT, Mms19P: n = 200, da>CAK, Mms19P: n = 100. SS was calculated using Fisher’s exact test. (PDF) [file pgen.1008913.s001.pdf]

**S3 Fig: *Mms19* is cell autonomously required to maintain normal cell numbers in MARCM clones**

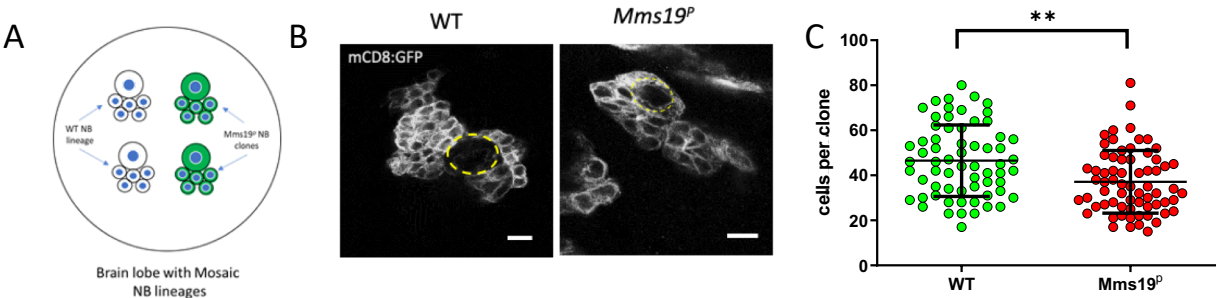

Supplement: S3 Fig — (A)-(C) In order to study cell cycle progression in a single NB lineage, we used the Mosaic Analysis with a Repressible Cell Marker (MARCM) technique [50]. This technique utilizes the UAS-GAL4-GAL80 system and the FLP-FRT recombination system. With this technique, a population of cells arising from the same progenitor can be specifically labeled. Additionally, the progenitor cell can carry a mutation along with a GFP marker. Defects in this cell, along with its progeny can be analyzed in an otherwise wild-type background. (B) MARCM clones were induced in NBs in 24hrs old larvae. These larvae were dissected after another 48hrs to determine the number of cells per clone in Mms19P and wild-type control clones. (C) The graph shows a significant reduction in the numbers of cells in mutant clones. SS was determined by an unpaired t-test (**P<0.01), scale = 5μm, n = 60 clones from each genotype. (PDF) [file pgen.1008913.s003.pdf]

**S4 Fig: *Mms19* is necessary for centrosomal localization of Msps in NBs**

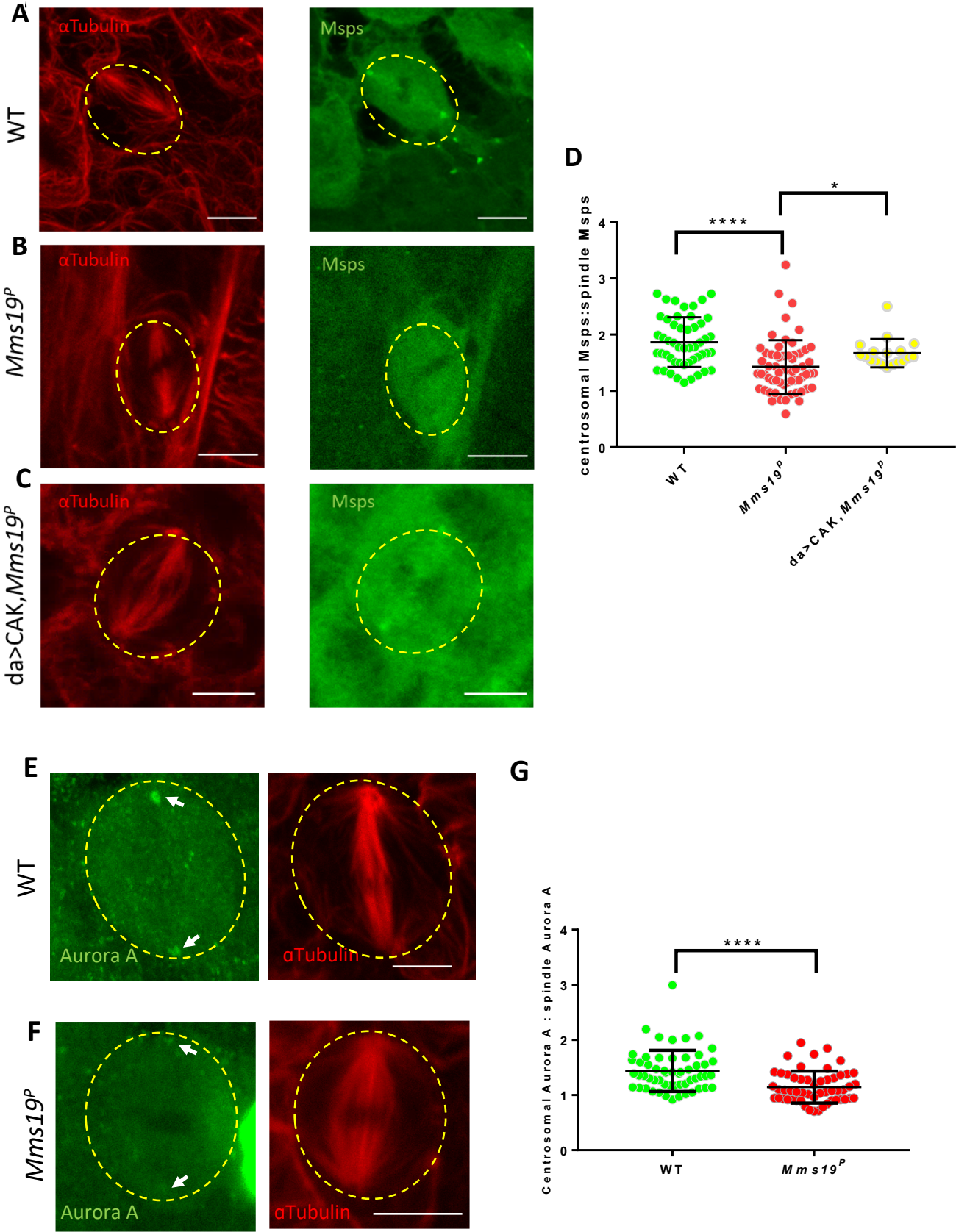

Supplement: S4 Fig — (A, C) WT and da>CAK, Mms19P NBs showing Msps localization on centrosomes and spindles. (B) In Mms19P NBs, Msps does not concentrate on centrosomes. (D) To quantify the centrosomal accumulation of Msps, an analysis similar to that done in Fig 6 was performed. SS was calculated using Kruskal-Wallis test, columns were compared using Dunn’s post test, ***(P<0.001), *(P<0.05), scale = 5μm. WT, n = 25 NBs; Mms19P, n = 30 NBs; da>CAK, Mms19P, n = 9 NBs, 2 experiments. (E) Aurora A signal is enriched in the spindle pole region in metaphasic WT NBs (indicated by arrows) but seems to be depleted from the spindle pole region of Mms19P NBs (F). (G) The scatter plot represents the ratio of the fluorescent intensity of Aurora A on the centrosome to the background fluorescent signal on the spindles. N = 28 cells per genotype, 2 experiments. Columns were compared using unpaired Students’ t-test, ****(P<0.0001). (PDF) [file pgen.1008913.s004.pdf]

**S5 Fig: MT assembly defects in *Mms19<sup>P</sup>* NBs and *Mms19::eGFP* localization in NBs and in neurons.**

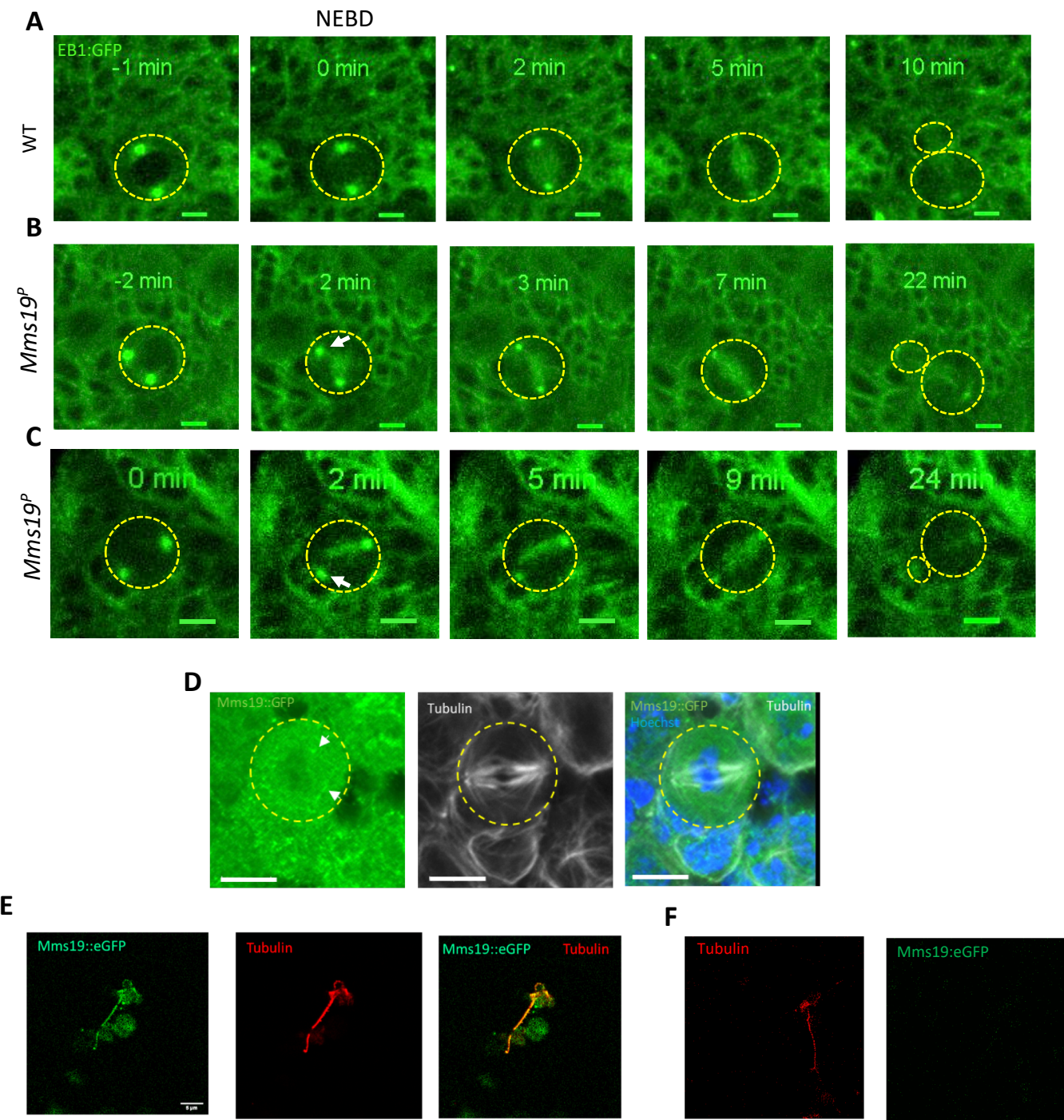

Supplement: S5 Fig — (A) WT NBs assemble a bipolar spindle 2–3 minutes after NEBD. On the other hand in some Mms19P NBs, (B, C) we observed a delay in MT assembly from one centrosome (indicated with arrows) and bipolar spindle assembly in these cells took on average 7–8 mins after NEBD. The centrosome which showed a delay in MT assembly was always inherited by the GMC. (D) Mms19 localization in NBs was determined by staining Mms19::eGFP, Mms19P NBs with anti-GFP antibodies. Although the Mms19::eGFP signal appears ubiquitous in the cytoplasm, we observe an enrichment on astral MTs (indicated by arrows). Scale = 5μm, n = 30 NBs, 2 experiments. (E) Neurons expressing Mms19:eGFP in the Mms19P background were stained with anti-GFP antibody to determine the localization of Mms19 in neurons. Mms19:eGFP signal co-localizes with α-Tubulin in the neurite. Scale = 5 μm, n = 30 neurons, 2 experiments. (F) No signal was observed in WT neurons stained with anti-GFP antibody, thus ruling out any non-specific signal by the anti-GFP antibody. (PDF) [file pgen.1008913.s005.pdf]

S6 Fig: Model for the function of Mms19 towards MTs

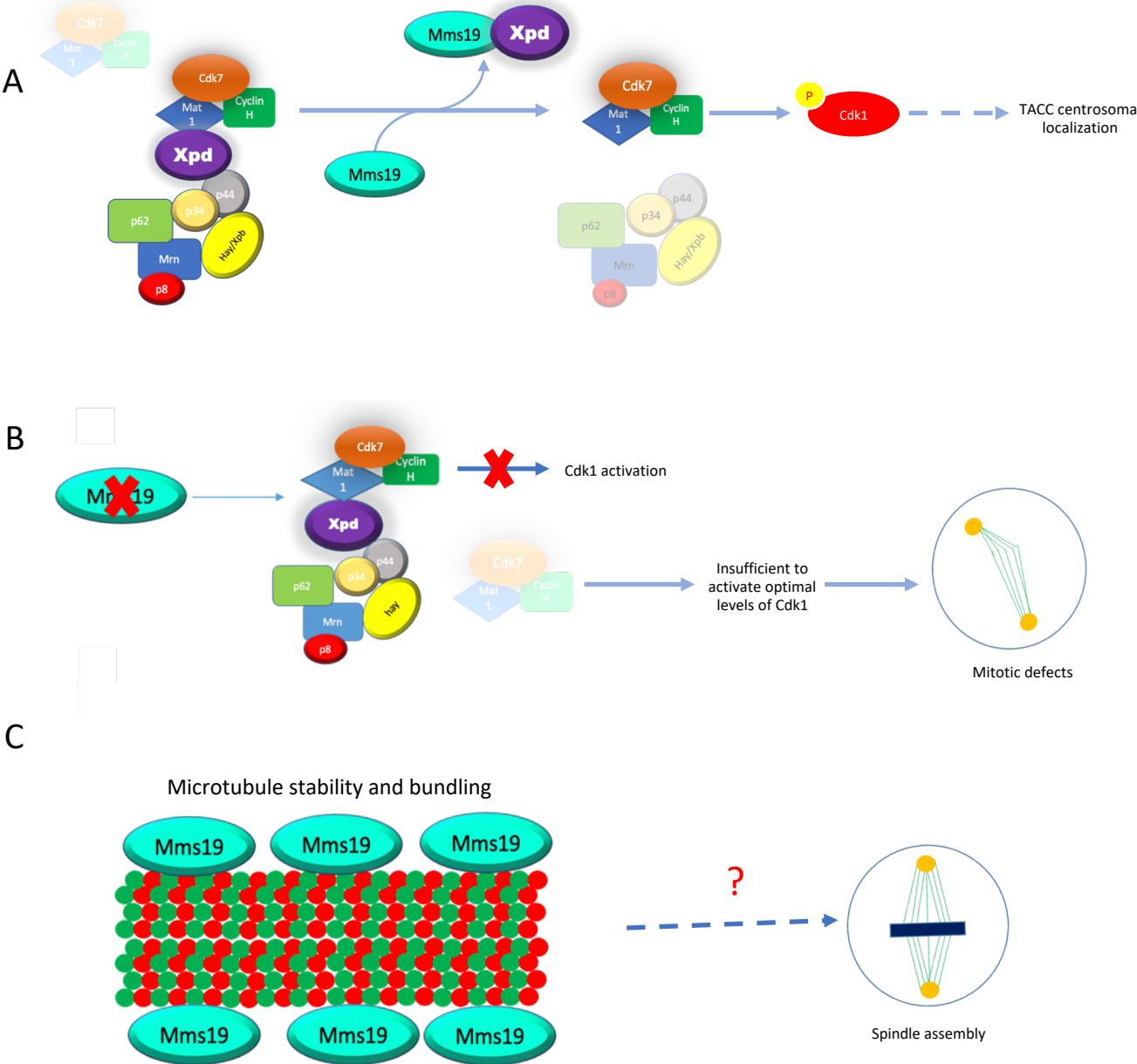

Supplement: S6 Fig — (A) During interphase, much of CAK is bound to the core TFIIH via Xpd. Even though basal levels of free CAK (shown above the TFIIH in faint colors) exist, this activity is below the required threshold to push cells into mitosis. During mitosis, Mms19 binds to Xpd, and thereby releases CAK and ensuring that sufficient CAK activity can drive mitosis via activation of Cdk1 and its downstream targets including Aurora A, TACC, and Msps. (B) Downregulation of Mms19 by mutations or knock-down allows Xpd to associate with CAK and core TFIIH, thereby targeting Cdk7 activity away from the mitotic targets and towards transcriptional targets like the PolII-CTD [10]. Though basal levels of CAK activity remain in this case, they are not able to bring about optimal activation of Cdk1, and therefore, when cells enter mitosis, this results in spindle assembly defects and mitotic delays. (C) Mms19 binds to MTs and appears to promote MT assembly, stability, and bundling. This novel activity of Mms19 could potentially contribute to establishing the extended MT structures in the mitotic spindle. (PDF) [file pgen.1008913.s006.pdf]
