# Supplementary material for: Mms19 promotes spindle microtubule assembly in Drosophila neural stem cells
Source: PLoS Genet. 2020 Nov 19;16(11):e1008913. doi: 10.1371/journal.pgen.1008913 (PMC7714366; doi:10.1371/journal.pgen.1008913)

S2 Fig: Phospho-Histone H3 (pH3) staining reveals a higher proportion of *Mms19^P^* NBs undergoing mitosis

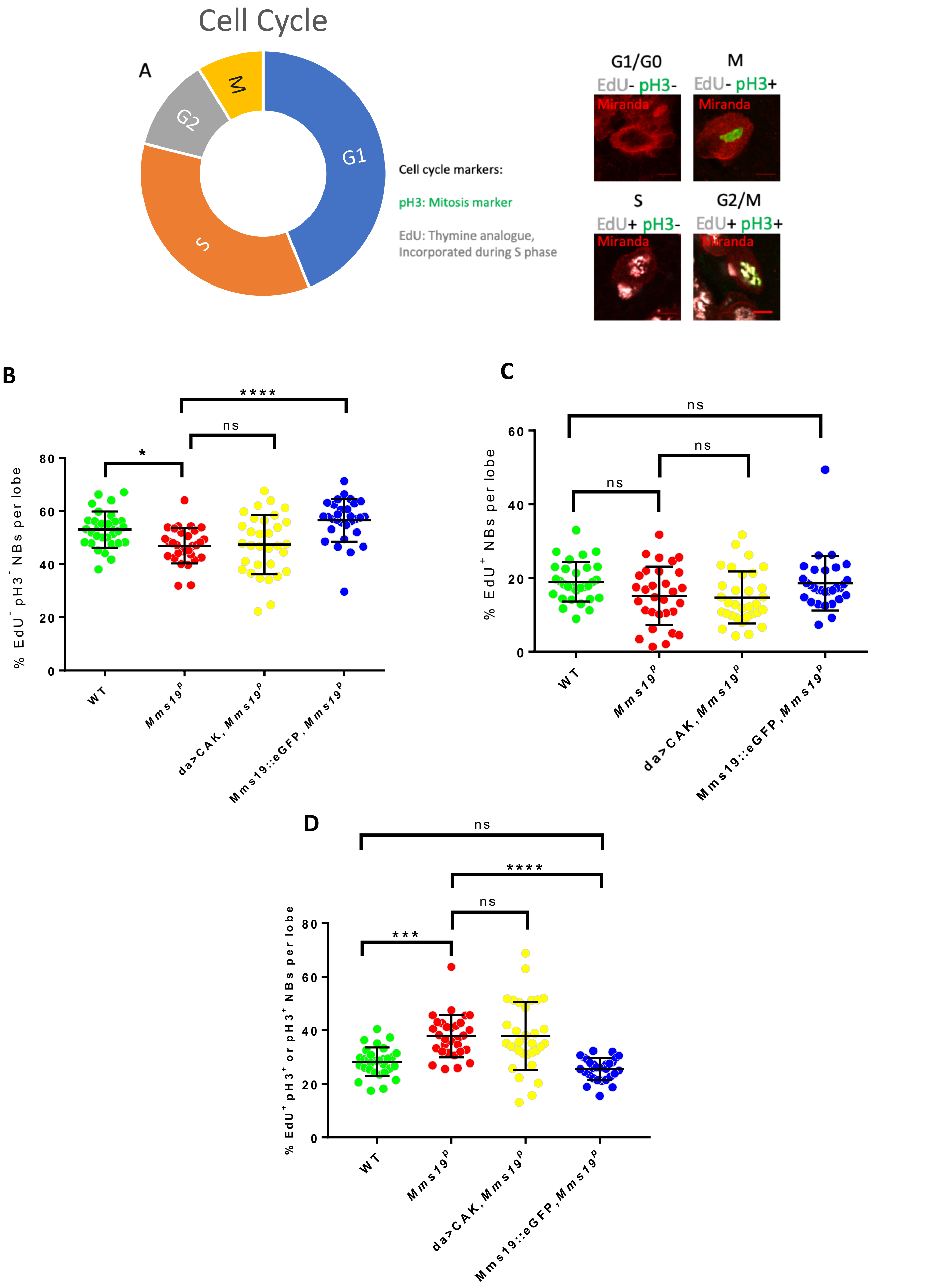

Supplement: S2 Fig — (A) NBs were classified into 1) G1/G0 phase if they did not stain for either EdU or pH3; 2) S phase if the NBs stained positively for EdU; 3) Mitotic phase for NBs staining positively for pH3 (independent of whether they stained for EdU or not). NBs in each phase were counted per brain lobe and this data was represented as percentage of total NBs in this lobe (e.g. if in one brain lobe 20 out of 100 NBs were EdU positive, then 20% cells were classified as in S phase). The percentages for each phase were compiled and compared per brain lobe across the 4 genotypes. Scatter dot plot charts represent the percentage of cells in (B) G1/G0 phase, (C) S phase and (D) mitotic phase. n = 30 brain lobes per genotype, experiments. SS was calculated using Kruskal-Wallis test, columns compared using Dunn’s post test, ****(P<0.0001), ***(P<0.001), *(P<0.05). Scale = 5μm (PDF) [file pgen.1008913.s002.pdf]
