# Supplementary material for: Mms19 promotes spindle microtubule assembly in Drosophila neural stem cells
Source: PLoS Genet. 2020 Nov 19;16(11):e1008913. doi: 10.1371/journal.pgen.1008913 (PMC7714366; doi:10.1371/journal.pgen.1008913)
Supplement: S1 Table — CIA proteins, which are already known to form a complex with Mms19, are highlighted in Red. Microtubule associated proteins are highlighted in Green. (PDF) [file pgen.1008913.s007.pdf]

S1 Table

In order to find novel Mms19-interacting proteins, we prepared protein extracts from adult flies expressing Mms19::eGFP and performed co-IP with anti-GFP antibody-coated beads. Protein complexes bound to the beads were then analyzed by Mass spectrometry. Simultaneously, we also performed co-IP and Mass spectrometry analysis for protein extracts prepared from wild type and Imp::GFP expressing flies to determine any non-specific binding to the antibody, beads or GFP. Amongst other interesting candidates, we found the Cytoplasmic Iron sulfur Assembly (CIA) proteins and some Microtubule associated proteins exclusively co-purifying with Mms19::eGFP. Tubulin on the other hand is quite abundant and was hence found in co-IPs of all extracts, although it was clearly enriched in the Mms19::eGFP extract compared to the wild type.

Abbreviations used:

PSM: Peptide Spectral Match, defines a potentially correct interpretation of a single fragment spectrum.

SpS: Spectrum Summation, counting of how many times one particular protein was identified by peptide spectral matches. Also referred to as spectrum counting.

PMSS: Protein Match Score Summation, all scores from peptide spectral matches to one particular protein are added up irrespective of whether a peptide was identified several times, with or without modifications. PMSS is sort of an extension of SpS, adding a spectral quality factor. The idea is that fragment spectra of peptides coming from high abundant proteins in the sample will result more often in good quality spectra than peptides from low abundant proteins. In the latter case there are less parent ions available for fragmentation which results in decreased fragment ion intensities or noisy spectra which will receive decreased scores.

The table lists proteins found to exclusively co-purify with Mms19::eGFP. CIA proteins, which are already known to form a complex with Mms19, are highlighted in Red. Microtubule associated proteins are highlighted in Green.

Protein	Accession no.	PMSS	Total coverage (%)	Total PSMs	Total Peptides
Putative elongator complex protein 1 (ELP1)	Q9VGK7	1078.27928	30.75	100	35
Probable cytosolic iron-sulfur protein assembly protein Ciao1	Q7K1Y4	795.474183	38.21	76	14
Probable cytosolic Fe-S cluster assembly factor CG17683	Q8SYS7	765.08696	46.54	77	20
Probable elongator complex protein 3	Q9VQZ6	637.901947	44.93	61	21
Cytoplasmic tRNA 2-thiolation protein 2	Q9VIV3	574.234751	51.36	52	17
CG6907, isoform B;	X2J4N6	554.880346	40.27	51	15
Importin subunit alpha	P52295	362.428368	34.1	34	12
Probable elongator complex protein 2 (ELP2)	Q7K4B3	359.419502	25.69	36	17
Cytoplasmic tRNA 2-thiolation protein 1	Q7JWW5	342.765471	36.44	33	10
Ribonucleoside-diphosphate reductase large subunit	P48591	342.380554	20.69	33	14
LD47396p;	Q9VHL2	329.163679	33.09	39	15
CG17374;	Q7PLB8	309.738944	8.06	38	18
MIP18 family protein CG7949	Q9VTC4	309.256955	64.1	28	7
CG6523-PA;	Q9VJZ6	302.20667	60.65	28	9
Anon-i1 protein;	Q24050	295.765288	48.47	28	11
DID;	Q9NH01	294.77212	34.66	24	8
Dynamin related protein 1, isoform A;	Q9VQE0	286.587794	17.14	28	11
Importin subunit beta	O18388	257.005417	15.27	25	10
T-complex protein 1 subunit delta	Q9VK69	252.435812	30.96	30	13
Cullin-associated NEDD8-dissociated protein 1	Q9VKY2	235.573965	11.38	26	13
D-Importin 7/RanBP7;	Q9VSD6	234.518326	9.34	26	8
26S proteasome non-ATPase regulatory subunit 11	Q7KLV9	231.276633	19.19	24	9
RuvB-like helicase 1 (Dpon)	Q9VH07	177.620244	26.1	17	8
FI07923p;	Q9VN44	170.520532	12.49	20	10
Ef1alpha-like factor, isoform A;	Q9VK85	164.386313	15.02	18	8
Proteasome subunit beta type-3	Q9XYN7	161.38841	28.78	14	5
Asparaginyl-tRNA synthetase, isoform A;	Q9V434	160.477915	13.62	16	7
Regulatory particle triple-A ATPase 4;	Q9W414	152.897841	27.96	16	10
Proteasome subunit alpha type-5	Q95083	151.799911	30.74	17	7
Coatomer subunit gamma (Gamma-COP) [ISOFORM C]	Q8I0G5_ISOFORM_C	150.822963	7.4	13	5
Proteasomal ubiquitin receptor ADRM1 homolog	Q7K2G1	149.996583	23.65	16	6
Exportin-2 (Exp2)	Q9XZU1	149.570563	9.44	15	7
V-type proton ATPase subunit H (V-ATPase subunit H)	Q9V3J1	140.253873	17.95	15	7

Chromatin assembly factor 1 subunit, isoform B;	E1JIL4	138.902673	9.56	13	4
Transport and golgi organization 7, isoform B;	A0A0B4LFB3	135.903285	18.09	18	7
Eukaryotic translation initiation factor 3 subunit C (eIF3c)	A1ZAX1	134.87918	9.12	17	8
Eukaryotic translation initiation factor 3 subunit F-1 (eIF3f-1)	Q9VN50	134.585454	17.14	14	4
Alpha-mannosidase	Q8IPB7	134.192405	7.13	14	5
Proteasome subunit beta type-1 [CHAIN 0]	P40304_CHAIN_0	133.196417	40.47	19	6
Uncharacterized protein CG16817	Q9VH95	129.659679	31.52	10	4
GH27579p;	Q9V3D8	128.576844	13.98	16	6
Probable glutamine--tRNA ligase (GlnRS)	Q9Y105	127.130684	7.58	12	5
Proteasome subunit alpha type-1	P12881	124.839184	30.82	15	7
Ubiquitin carboxyl-terminal hydrolase	Q9XZ61	123.734264	18.83	16	6
Eip55E, isoform A;	Q7JXZ2	120.938782	18.83	15	6
Dynein heavy chain, cytoplasmic (DYHC) [ISOFORM C]	P37276_ISOFORM_C	120.90723	1.96	13	8
Leukotriene A(4) hydrolase (LTA-4 hydrolase)	Q9VJ39	120.257714	17.62	13	8
CG4847, isoform A;	Q7K5N8	117.236533	21.79	16	5
Probable 3-hydroxyisobutyrate dehydrogenase, mitochondrial (HIBADH) [CHAIN 0]	Q9V8M5_CHAIN_0	116.729559	17.73	12	4
GH11341p;	Q9V3Z4	115.20378	5.78	15	3
DNA polymerase	Q9VCN1	113.17361	3.62	12	7
LD24721p;	Q9VJD4	111.577302	12.08	14	3
FI22042p1;	T2GFI6	110.823946	5.34	13	5
BcDNA.GH02678;	Q9Y162	110.087038	11.31	11	5
Protein krasavietz (Decp) (eIF5C domain-containing protein)	Q9VNE2	109.953585	8.77	15	4
Eukaryotic translation initiation factor 3 subunit E (eIF3e)	O77410	109.575072	14.02	11	6
Protein purity of essence	Q9VLT5	108.664591	2.03	12	9
CG6453, isoform A;	Q9VJD1	108.035339	6.75	15	4
26S proteasome non-ATPase regulatory subunit 14	Q9V3H2	107.405637	25.32	14	6
Coatomer subunit beta (Beta-COP)	P45437	104.684205	5.29	11	4
Stathmin-1;	Q9GQR6	104.133146	14.53	9	3
CG12244-PA;	O62602	103.392352	12.87	11	4
Calcium-binding protein 1, isoform A;	Q9V438	103.241084	13.63	13	5
CG2918, isoform B;	O46067	102.824666	6.93	11	5
Ubiquitin carboxyl-terminal hydrolase	Q9VKZ8	102.635563	8	9	3
Probable 26S proteasome non-ATPase regulatory subunit 3 (26S proteasome subunit S3) (DOX-A2)	P25161	102.195153	8.1	15	4
SD25413p;	Q86NM8	100.882168	9	11	3

Amidophosphoribosyltransferase (ATase)	Q9VRZ1	100.861053	13.62	14	6
Serine/threonine-protein phosphatase PP2A	P23696	99.301938	19.74	11	5
Proteasome subunit beta type-4	Q9VNA5	97.072653	11.94	13	3
Serine/threonine-protein phosphatase 2A 55 kDa regulatory subunit B	C8VV30	93.750053	12.19	11	4
Peptidoglycan-recognition protein SB1 [CHAIN 0]	Q70PY2_CHAIN_0	92.932968	22.29	9	4
Ribosomal protein S29, isoform B;	A0A0C4DHE8	92.31717	50	15	3
AT11715p;	Q8MR83	91.962815	7.62	9	5
CG8778;	A1Z934	90.897119	11.71	11	3
CG1707;	A1Z6X6	90.626258	21.59	14	3
Ubiquitin carboxyl-terminal hydrolase	Q9VZU7	90.607458	9.43	12	7
LD20211p;	Q9W0S7	88.912386	6.26	11	5
Kinesin heavy chain	P17210	88.485361	6.15	11	5
CG2767;	Q9VHX4	86.79088	12.77	10	4
CG10652-PA, isoform A;	Q9VJ19	85.436545	27.03	11	3
AT25839p;	Q5U0U9	83.524201	2.82	8	3
RH58777p;	Q8SXW1	82.654699	21.43	14	2
CG7911, isoform A;	Q9VAC4	82.138146	27.56	8	3
CG12171;	Q9VNF3	82.129288	14.4	8	3
Serine/threonine-protein phosphatase alpha-3 isoform	Q05547	80.845538	9.6	10	3
CG3226-PA;	Q9W3Y3	79.976904	16.09	8	3
Tyrosine--tRNA ligase	Q9VV60	78.389427	10.67	8	5
CG9436;	Q7JVH6	77.762115	17.04	10	5
Eukaryotic translation initiation factor 3 subunit H (eIF3h)	Q9U9Q4	77.167678	10.65	9	3
CG1041, isoform B;	Q0KIA8	76.669146	7.46	11	4
Regulatory particle non-ATPase 10, isoform B;	M9PIG8	76.52857	8.33	9	3
Odorant-binding protein 56e, isoform B;	A0A0B4LFW3	74.809926	23.66	9	3
MIP08680p;	Q9VDY8	74.174086	24.26	8	4
Dynamin [ISOFORM 2]	P27619_ISOFORM_2	72.157736	6.51	8	4
26S proteasome regulatory complex subunit p39A;	Q7KMP8	71.026223	12.3	9	4
Peptidyl-prolyl cis-trans isomerase	Q9VGK3	70.85612	18.84	8	2
CG12512;	Q9VMR6	70.80174	7.42	12	4
General odorant-binding protein 99a [CHAIN 0]	Q9VAJ4_CHAIN_0	70.008403	21.43	14	3
6-phosphogluconate dehydrogenase, decarboxylating	P41572	69.066395	13.31	12	5
Eukaryotic initiation factor 4B, isoform E;	Q7PLL1	68.644589	14.65	11	4
Protein NASP homolog	Q9I7K6	66.902889	10.37	6	3
CG2862, isoform B;	Q86BQ4	66.827759	28.57	8	3
Dihydropteridine reductase;	Q9VSU6	65.57742	11.49	5	2
CG13072-PA;	Q9VUZ8	64.853286	17.29	6	2
Carboxypeptidase [CHAIN 0]	Q9VDT5_CHAIN_0	64.169304	5.7	7	2

Gag protein;	Q9NHF8	62.915404	6.43	10	2
CG3663;	Q9W127	61.90281	13.46	5	2
CG15261-PA;	Q9V3W0	60.683397	30.43	8	3
MIP16878p;	D3PFE0	60.21821	4.35	7	4
40S ribosomal protein S5b	Q9VFE4	59.94444	14.35	9	4
Myosin-2 essential light chain	P54357	59.22928	27.21	5	3
NAT1 ortholog, isoform A;	Q9VWI2	58.946433	3.03	7	2
Coproporphyrinogen oxidase, isoform B;	X2J9R2	58.26964	5.64	10	2
DNA replication licensing factor Mcm6 (DmMCM6)	Q9V461	58.103871	4.65	6	3
Mutant tan;	Q9W369	57.867012	6.72	5	2
40S ribosomal protein S27	Q9VBU9	56.930696	22.62	10	2
Dihydropyrimidine dehydrogenase;	Q9W374	56.391749	3.3	6	3
V-type proton ATPase subunit D 1 (V-ATPase subunit D 1)	Q9V7D2	56.318904	9.35	10	2
SD06613p;	Q960D3	55.989689	8.5	8	3
COP9 signalosome complex subunit 7 (Dch7) (Signalosome subunit 7)	Q9V4S8	55.844796	13.67	6	3
CG3603, isoform B;	Q9W4U2	54.727119	10.44	5	2
Deoxyuridine triphosphatase, isoform B;	Q8IPB1	54.204969	17.24	6	3
Ribosomal protein S5a, isoform B;	X2JKU5	54.12625	17.98	10	5
Glutathione synthetase (GSH-S)	Q8IQZ1	54.012329	5.91	5	2
Female-specific independent of transformer;	Q9VD66	53.872528	19.83	5	2
Phospholipase A2 activator protein, isoform A;	Q9VPY2	53.862268	2.92	8	2
ADP-ribosylation factor 1 [CHAIN 0]	P61209_CHAIN_0	53.852292	9.94	5	3
Gag protein;	Q0GA05	53.755813	3.99	6	2
CG1943, isoform A;	Q9VI56	53.322602	31.36	7	2
LD43045p;	Q8SWV7	52.947145	1.56	6	2
Accessory gland protein Acp62F [CHAIN 0]	O46202_CHAIN_0	52.767269	31.87	8	3
CG34215;	A8DY49	52.642595	20.19	8	2
Cystathionine beta-synthase	Q9VRD9	51.33755	4.41	5	2
CG3835, isoform A;	Q7K511	51.230366	8.07	5	3
GM13889p;	Q8MRJ8	51.20354	6.81	10	2
CG6330, isoform B;	Q8IMQ8	50.807596	5.48	6	2
CG17514, isoform A;	Q7PLL6	50.662459	1.14	9	3
CG7322, isoform B;	Q9VWP2	50.173374	13.22	7	3
Burgundy, isoform B;	Q9VIE7	50.143559	2.78	7	2
Sperm-specific dynein intermediate chain 1, isoform C;	M9NE47	50.055616	7.5	7	3
Kinesin light chain, isoform B;	M9PF24	49.874176	13.19	6	5
Tim8, isoform B;	X2JB87	49.338077	30.68	4	2
LD16419p;	Q9VW59	49.054259	11.44	11	3
CG8209;	Q9VSC5	48.93248	9.67	4	2
Replication protein A 70 kDa DNA-binding subunit (RP-A p70) (RF-A protein 1) (DmRPA1)	Q24492	47.526955	3.32	4	2

Serine protease inhibitor (Serpin-5);	Q9U1I4	47.265776	5.62	6	2
CG16826, isoform A;	Q9VK00	47.078998	12.61	5	3
CG9273 protein;	Q9VIH1	46.879655	12.6	5	2
LD22082p;	Q9VLQ9	46.838306	6.24	5	3
DNA replication licensing factor Mcm3 (DmMCM3)	Q9XYU1	46.585268	2.32	5	2
LP08082p;	Q9VGP4	46.372649	4.13	5	3
CG10126, isoform A;	Q9VG23	46.363266	13.49	4	2
MIP14975p;	D0Z726	45.705535	24.72	5	2
Smt3 activating enzyme 2;	Q9VSD9	45.602776	4	5	2
Methionine aminopeptidase 2 (MAP 2) (MetAP 2)	Q9VL89	45.36067	4.46	4	2
CG1236;	Q8I725	45.028941	6.34	5	2
CG11878;	Q9VBT8	44.92775	6.19	4	2
GH08387p;	Q9VHA2	43.575072	18.43	6	3
Small nuclear ribonucleoprotein-associated protein B (snRNP-B) (Sm-B) (SmB)	Q05856	43.555329	11.56	7	2
RE07481p;	Q8SXL6	43.490619	9.54	4	2
Methylthioadenosine phosphorylase ortholog, isoform C;	A0A0B4LFH0	43.271402	9.69	7	3
BcDNA.HL02693;	Q9V3N7	43.157532	5.6	9	3
GH14654p;	Q7KK90	42.940316	7.14	5	2
CG5590;	Q9VB10	42.718398	5.83	6	2
Probable pseudouridine-5'-phosphatase (5'-PsiMPase)	Q94529	42.145333	11.26	6	3
CG6767, isoform A;	Q9VT32	41.631079	9.43	9	3
Prefoldin subunit 3	Q9VGP6	40.7714	13.92	5	3
Glucose-6-phosphate 1-dehydrogenase (G6PD)	P12646	40.739479	4.01	9	2
Serine/threonine-protein phosphatase	Q8SWU9	40.709004	6.06	4	2
Hsp90-related protein TRAP1;	Q7KNF3	40.652344	6.12	5	3
Groucho, isoform I;	A0A0B4KH09	40.451027	3.15	5	2
39 kDa FK506-binding nuclear protein (PPIase)	P54397	39.425323	5.88	4	2
Alpha-amylase B [CHAIN 0]	P81641_CHAIN_0	39.249233	6.3	3	2
Inositol-3-phosphate synthase (MIP synthase) (IPS) (MI-1-P synthase)	O97477	39.013919	9.91	4	3
RE04051p;	Q5BI95	38.307076	3.68	4	2
Ubiquitin-like protein activating enzyme N subunit;	Q7KJV5	38.297655	5.93	5	2
AT05114p;	Q8MRS4	38.212791	1.98	4	2
Cytochrome b-c1 complex subunit Rieske, mitochondrial	Q9VQ29	38.120393	10.87	8	2
CG11999;	Q9VNA3	37.985766	14.81	5	3
CG5012;	Q2XYH0	37.690651	23.67	7	2
CG2493, isoform A;	Q9VIM0	37.361102	5.89	5	2
ATP-dependent RNA helicase vasa, isoform A	P09052	37.137186	3.48	7	2
Nuclear migration protein NudC;	Q9VVA6	36.339406	5.12	5	2

Endonuclease G inhibitor, isoform A;	Q9V3V9	35.36594	5.85	9	2
Adenylosuccinate synthetase (AMPSase) (AdSS)	F0JAP3	35.208644	9.23	5	4
DNA damage-binding protein 1 (D-DDB1)	Q9XYZ5	34.877239	1.58	4	2
RE50216p;	Q8IGP9	34.626232	13.64	6	3
CG10672;	Q9VRJ4	34.517959	5.68	4	2
CG1774, isoform A;	Q9V9W4	34.506729	6.78	5	2
IP17959p;	A4IJ71	34.062606	7.73	4	2
Glutathione S transferase E6;	A1ZB71	33.548655	12.16	3	2
Protein slender lobes	Q8INM3	33.367991	1.55	4	2
Protein singed	Q24524	33.338293	3.91	4	2
SH3 domain-binding glutamic acid-rich protein homolog (SH3BGR protein)	Q9NFP5	33.222539	11.39	7	2
CG10407, isoform A;	Q9VEZ6	33.062111	15.83	4	3
Histone H2A	Q4AB57	32.506428	13.71	10	2
Myosin regulatory light chain sqh (MRLC-C)	P40423	32.456961	21.26	5	4
CG3714, isoform G;	M9PC31	32.42235	5.27	7	3
CG10964-PA;	Q9W3H4	32.175885	8.5	6	2
Collapsin response mediator protein;	Q8IPQ2	32.033792	5.05	4	3
Profilin	P25843	31.648317	14.29	5	2
CG14407;	Q8SXQ5	30.723439	16.98	7	2
Lissencephaly-1, isoform G;	A0A0B4K812	30.652584	6.57	3	2
Probable arginine--tRNA ligase, cytoplasmic (ArgRS)	Q9VXN4	29.109661	4.81	3	3
CG4199, isoform A;	O77266	29.095694	3.44	3	2
Globin;	Q9U1K3	29.091126	21.57	5	3
LD01551p;	D1Z373	28.998582	4.79	3	2
CG9547;	Q9VMC6	28.45929	7.88	6	2
Proteasome subunit beta type	Q9NHX7	27.986975	8.51	3	2
Sex-regulated protein janus-A [ISOFORM A]	P20348_ISOFORM_A	27.647884	25.21	3	2
Glycylpeptide N-tetradecanoyltransferase (NMT)	O61613	27.345608	4.66	3	2
Thiolester containing protein II, isoform E;	A0ZWS9	27.083716	2.93	5	3
Neural Lazarillo;	Q9NAZ4	26.911862	11.61	3	2
Angiotensin-converting enzyme-related protein [CHAIN 0]	Q9VLJ6_CHAIN_0	26.590681	4.28	4	2
Proteasome subunit alpha type-2	P40301	26.174957	13.68	3	2
DNA replication licensing factor Mcm2 (DmMCM2)	P49735	25.48558	2.48	3	2
Dynein light intermediate chain, isoform A;	Q9VZ20	24.976181	4.67	3	2
Aldose 1-epimerase	Q9VRU1	24.97472	6.04	3	2
Putative Sec23 protein;	Q9NFN9	24.965424	4.29	3	3
CG9360-PA;	Q9VYU9	24.961089	7.57	6	2
cAMP-dependent protein kinase catalytic subunit (PKA C) [CHAIN 0]	P12370_CHAIN_0	24.815355	9.94	4	3

CG10623 protein;	Q9VJ31	24.674937	5.74	7	2
Nuclear protein localization protein 4 homolog [ISOFORM B]	Q9VBP9_ISOFORM_B	24.453279	3.37	3	2
CG13993, isoform B;	Q9VMH8	24.317949	14.29	4	2
Methylthioribose-1-phosphate isomerase (M1Pi) (MTR-1-P isomerase)	Q9V9X4	23.159481	5.77	3	2
GM14873p;	Q8T9H2	22.649018	7.24	3	2
Glutathione S transferase O3;	Q9VSL2	22.393017	12.45	4	3
Proliferating cell nuclear antigen (PCNA)	P17917	22.331198	6.15	3	2
CG5844;	Q9VG69	22.227807	7.41	4	2
CG9953;	Q9VS02	21.98977	8.27	5	3
GH10029p;	Q7K571	21.58354	3.74	2	2
AT30379p;	F0JAL1	20.185758	8.92	3	2
Probable dynactin subunit 2 (Dynamitin)	Q7K2D2	19.713939	6.58	2	2
Protein translation factor SUI1 homolog	Q9VZS3	19.59736	17.27	3	2
RH50948p;	F0JAK0	19.58742	10.95	2	2
Chorion protein S36 [CHAIN 0]	P07182_CHAIN_0	19.122952	11.74	2	2
Acidic leucine-rich nuclear phosphoprotein 32 family member A	Q9V895	18.350596	12.64	3	3
CG32473, isoform A;	Q9VFW7	18.284285	2.87	4	3
CG31549, isoform A;	Q8SX57	18.281097	8.56	2	2
LD37855p;	Q8T025	18.093456	8.49	2	2
Sorting nexin 1, isoform B;	X2J8I7	18.087694	5.48	2	2
Enolase-phosphatase E1	Q9VN95	17.962747	10.55	2	2
26S proteasome regulatory complex subunit p30;	Q9V436	17.500526	12.12	2	2
Probable protein phosphatase CG10417	Q7K4Q5	17.202213	5.59	2	2
CG31472, isoform B;	Q7KSW3	16.673093	8.02	2	2
Succinyl-CoA:3-ketoacid-coenzyme A transferase	M9PGX7	16.480237	4.67	2	2
LD45279p;	Q960M5	16.18657	6.9	2	2
CG13175;	A1Z8U7	15.853459	20.57	2	2
ALG-2 interacting protein X;	Q9VB05	15.070043	1.79	2	2
Armadillo repeat-containing protein 6 homolog	Q7K486	14.144357	4.09	2	2
