# Supplementary material for: Mms19 promotes spindle microtubule assembly in Drosophila neural stem cells
Source: PLoS Genet. 2020 Nov 19;16(11):e1008913. doi: 10.1371/journal.pgen.1008913 (PMC7714366; doi:10.1371/journal.pgen.1008913)
Supplement: S2 Table — These include different isoforms of α-and βtubulin that were identified in all extracts. However, the PMSS scores are higher for the tubulin isoforms immuno-precipitating from the Mms19::eGFP extract. This indicates that tubulin was enriched in the Mms19::eGFP fraction compared to the wild-type control. (PDF) [file pgen.1008913.s008.pdf]

## S2 Table

In order to find novel Mms19-interacting proteins, we prepared protein extracts from adult flies expressing Mms19::eGFP and performed co-IP with anti-GFP antibody-coated beads. Protein complexes bound to the beads were then analyzed by Mass spectrometry. Simultaneously, we also performed co-IP and Mass spectrometry analysis for protein extracts prepared from wild type and Imp::GFP expressing flies to determine any non-specific binding to the antibody, beads or GFP. Amongst other interesting candidates, we found the Cytoplasmic Iron sulfur Assembly (CIA) proteins and some Microtubule associated proteins exclusively co-purifying with Mms19::eGFP. Tubulin on the other hand is quite abundant and was hence found in co-IPs of all extracts, although it was clearly enriched in the Mms19::eGFP extract compared to the wild type.

**Abbreviations used:**

**PSM:** Peptide Spectral Match, defines a potentially correct interpretation of a single fragment spectrum.

**SpS:** Spectrum Summation, counting of how many times one particular protein was identified by peptide spectral matches. Also referred to as spectrum counting.

**PMSS:** Protein Match Score Summation, all scores from peptide spectral matches to one particular protein are added up irrespective of whether a peptide was identified several times, with or without modifications. PMSS is sort of an extension of SpS, adding a spectral quality factor. The idea is that fragment spectra of peptides coming from high abundant proteins in the sample will result more often in good quality spectra than peptides from low abundant proteins. In the latter case there are less parent ions available for fragmentation which results in decreased fragment ion intensities or noisy spectra which will receive decreased scores.

Proteins that bound to the anti-GFP antibody coated beads from all three fly extracts are listed. These include different isoforms of α-and βtubulin that were identified in all extracts. However, the PMSS scores are higher for the tubulin isoforms immuno-precipitating from the

Mms19::eGFP extract. This indicates that tubulin was enriched in the Mms19::eGFP fraction compared to the wild-type control.

| **Mms19::eGFP** | **Imp::GFP** | **wild type** | | |
|---|---|---|---|---|
| **PMSS** | **PMSS** | **PMSS** | **Accession no.** | **Description** |
| 3340,474185 | 1760,246969 | 3319,12355 | Q9V496 | Apolipophorin-1 |
| 2338,436802 | 1132,387337 | 860,510753 | Q9XTL9 | Glycogen phosphorylase |
| 1736,689022 | 1489,230637 | 1145,051713 | P11147 | Heat shock 70 kDa protein cognate 4 |
| 1580,213491 | 457,537864 | 375,495604 | A0A0B4JCW4 | Succinyl-CoA ligase subunit beta |
| 1568,201598 | 969,73428 | 700,424552 | Q9VQL7 | CG3523, isoform A; |
| 1525,616386 | 1322,298852 | 1071,734112 | Q9VIE8 | Aconitase, isoform B; |
| 1477,657455 | 809,006151 | 629,803314 | P02828 | Heat shock protein 83 |
| 1473,730409 | 1241,958002 | 1273,768862 | A8JNU6 | Neural conserved at 73EF, isoform I; |
| 1434,449869 | 1245,137804 | 1273,922818 | Q8IQQ0 | CG11661-PF, isoform F; |
| 1282,669993 | 1448,561123 | 2313,613173 | P29845 | Heat shock 70 kDa protein cognate 5 |
| 1279,648707 | 914,950395 | 496,615158 | O62619_ISOFORM_B | Pyruvate kinase (PK) [ISOFORM B] |
| 1277,714693 | 755,526014 | 695,929175 | Q9Y119 | BcDNA.GH08860; |
| 1239,287804 | 722,1279 | 721,908177 | Q24560 | Tubulin beta-1 chain |
| 1225,117964 | 1046,393098 | 929,133523 | Q9VWH4_ISOFORM_A | Probable isocitrate dehydrogenase [NAD] subunit alpha, mitochondrial [ISOFORM A] |
| 1200,406235 | 990,974693 | 732,275752 | P15007_ISOFORM_A | Enolase [ISOFORM A] |
| 1198,66663 | 528,967521 | 678,343753 | Q7KN97 | Pyruvate carboxylase |
| 1197,459537 | 1217,540409 | 943,375413 | P07764_CHAIN_0 | Fructose-bisphosphate aldolase [CHAIN 0] |
| 1152,949151 | 779,705487 | 689,007454 | A8JRB8 | CG5028, isoform C; |
| 1102,200137 | 1232,455907 | 1031,947437 | P48610 | Arginine kinase (AK) |
| 1089,122707 | 107,233987 | 43,519561 | Q9VVA4 | CG9674, isoform A; |
| 1069,543996 | 209,117725 | 406,797086 | P06607_CHAIN_0 | Vitellogenin-3 [CHAIN 0] |
| 1052,951539 | 835,027094 | 740,363674 | P13706_ISOFORM_GPDH-1 | Glycerol-3-phosphate dehydrogenase [NAD(+)], cytoplasmic (GPD-C) (GPDH-C) [ISOFORM GPDH-1] |
| 954,803201 | 683,463264 | 808,711421 | P29844_CHAIN_0 | Heat shock 70 kDa protein cognate 3 [CHAIN 0] |
| 938,084065 | 367,737896 | 568,771445 | O97355_CHAIN_0 | Transferrin; [CHAIN 0] |
| 938,035475 | 367,494263 | 568,724755 | Q9VWV6 | Transferrin 1, isoform A; |
| 930,259422 | 619,093163 | 965,34448 | Q9V496_CHAIN_0 | Apolipophorin-1 [CHAIN 0] |
| 928,154207 | 913,317461 | 893,912385 | P10987_CHAIN_0 | Actin-5C [CHAIN 0] |
| 909,552723 | 895,697465 | 893,912385 | P02572_CHAIN_0 | Actin-42A [CHAIN 0] |
| 887,944607 | 399,718625 | 396,061267 | Q9VQL6 | GH22259p; |
| 843,845255 | 844,999776 | 863,219939 | E1JGP0 | LD04994p1; |
| 840,187809 | 864,273063 | 861,027289 | P83967_CHAIN_0 | Actin, indirect flight muscle [CHAIN 0] |
| 812,147361 | 564,537549 | 543,117976 | Q9VHN7 | CG8036, isoform B; |

| | | | | |
|---|---|---|---|---|
| 805,037722 | 643,554497 | 743,411407 | P07486 | Glyceraldehyde-3-phosphate dehydrogenase 1 (GAPDH I) |
| 802,581885 | 209,044843 | 180,545791 | Q7KN62 | Transitional endoplasmic reticulum ATPase TER94 |
| 765,8618 | 458,687046 | 499,194471 | K7WQ63 | Alpha tubulin 84B; |
| 757,785733 | 426,91701 | 297,805568 | Q9VAX7 | Beta-Tubulin at 97EF, isoform C; |
| 754,068405 | 495,820583 | 470,455031 | Q7KSU6 | CG8036, isoform D; |
| 730,794767 | 441,400858 | 598,304305 | P54385 | Glutamate dehydrogenase, mitochondrial (GDH) |
| 709,334965 | 341,83072 | 669,872094 | Q05825_CHAIN_0 | ATP synthase subunit beta, mitochondrial [CHAIN 0] |
| 696,814119 | 399,911808 | 562,813195 | P54385_ISOFORM_C | Glutamate dehydrogenase, mitochondrial (GDH) [ISOFORM C] |
| 691,824972 | 162,347141 | 336,878562 | P02843_CHAIN_0 | Vitellogenin-1 [CHAIN 0] |
| 678,123474 | 468,11282 | 355,299057 | Q9VD58 | CG6439, isoform A; |
| 669,720535 | 535,284987 | 566,720804 | P07487 | Glyceraldehyde-3-phosphate dehydrogenase 2 (GAPDH II) |
| 660,20129 | 539,027804 | 545,207249 | Q9VEB1 | IP09655p; |
| 652,071983 | 671,635744 | 693,002957 | P02574_CHAIN_0 | Actin, larval muscle [CHAIN 0] |
| 630,94309 | 482,810202 | 419,917954 | P13060_CHAIN_0 | Elongation factor 2 (EF-2) [CHAIN 0] |
| 628,324565 | 485,911971 | 843,455002 | P35381 | ATP synthase subunit alpha, mitochondrial |
| 614,116672 | 470,848176 | 831,290782 | P35381_CHAIN_0 | ATP synthase subunit alpha, mitochondrial [CHAIN 0] |
| 610,870086 | 393,877287 | 269,39954 | P52029 | Glucose-6-phosphate isomerase (GPI) (PGI) (PHI) |
| 606,050144 | 521,318705 | 305,940026 | P08736 | Elongation factor 1-alpha 1 (EF-1-alpha-1) |
| 603,294766 | 385,063748 | 334,06024 | Q01604 | Phosphoglycerate kinase |
| 590,936461 | 220,995425 | 88,273986 | X2JGG6 | Heat shock protein 26, isoform B; |
| 588,628543 | 593,892996 | 399,633664 | Q8MSI2 | GH15296p; |
| 573,635995 | 507,635231 | 843,767172 | Q9VGQ1 | CG5214; |
| 572,136484 | 60,753319 | 78,597035 | A0A0B4LGC0 | Tripeptidyl-peptidase II, isoform F; |
| 558,556411 | 985,587584 | 186,758747 | P23128 | Putative ATP-dependent RNA helicase me31b |
| 557,520077 | 250,898145 | 204,266761 | O02649 | 60 kDa heat shock protein, mitochondrial (HSP-60) |
| 555,583052 | 413,024144 | 302,541871 | Q7M3J9 | Tubulin beta-3 chain; |
| 546,792926 | 479,033645 | 282,925741 | Q9VVL7 | Dihydrolipoyl dehydrogenase |
| 537,31862 | 108,439634 | 272,981129 | X2JC31 | Clathrin heavy chain |
| 532,755873 | 62,062701 | 49,279671 | Q9VXQ5 | GH13725p; |
| 528,648854 | 423,142875 | 87,003597 | Q9V3R1_CHAIN_0 | Accessory gland protein Acp36DE [CHAIN 0] |
| 528,374836 | 495,003968 | 318,071898 | Q94522 | Succinyl-CoA ligase [ADP/GDP-forming] subunit alpha, mitochondrial (SCS-alpha) |
| 526,378346 | 395,636339 | 548,041159 | Q9VM14 | Acetyltransferase component of pyruvate dehydrogenase complex |
| 519,78102 | 177,046382 | 215,932128 | A4V0N4 | MIP16230p; |
| 518,154928 | 141,210537 | 106,942113 | P48605 | T-complex protein 1 subunit gamma (TCP-1-gamma) |
| 513,821774 | 307,239037 | 90,622494 | X2JC82 | Heat shock protein 27, isoform B; |
| 511,673697 | 352,590314 | 190,708494 | A1Z9E3 | Elongation factor Tu |
| 506,709509 | 102,642997 | 25,404171 | A1Z8U4 | T-complex chaperonin 5, isoform B; |

| | | | | |
|---|---|---|---|---|
| 506,575442 | 466,892152 | 327,933406 | Q9VNW6 | CG7470, isoform A; |
| 501,015257 | 109,038707 | 28,124164 | Q9VW54 | 26S proteasome regulatory complex subunit p97; |
| 500,097156 | 248,612775 | 59,415885 | Q9VPN5 | CG2720-PA; |
| 499,455826 | 204,49371 | 202,418377 | P31409 | V-type proton ATPase subunit B (V-ATPase subunit B) |
| 499,299002 | 320,378922 | 266,190253 | X2JGP4_CHAIN_0 | Protein disulfide-isomerase [CHAIN 0] |
| 493,236467 | 299,913378 | 132,525202 | P92177_ISOFORM_C | 14-3-3 protein epsilon [ISOFORM C] |
| 490,747059 | 523,855811 | 545,462691 | Q9W4H6 | Pyruvate dehydrogenase E1 component subunit alpha |
| 487,579736 | 404,516109 | 234,421587 | P05303 | Elongation factor 1-alpha 2 (EF-1-alpha-2) |
| 485,753735 | 270,636798 | 329,457982 | Q9W401 | Probable citrate synthase, mitochondrial |
| 483,094317 | 326,735909 | 444,406945 | Q7K5K3 | CG11876, isoform A; |
| 478,443563 | 327,598144 | 195,987221 | E8NH18 | RH27654p; |
| 475,097115 | 410,673406 | 137,761549 | Q8IPE8 | CG4389-PB, isoform B; |
| 472,722423 | 254,694491 | 312,524529 | A1ZBJ2 | CG7461, isoform B; |
| 465,948321 | 234,285165 | 599,268163 | P09180 | 60S ribosomal protein L4 |
| 465,119985 | 441,732311 | 288,970031 | P17336 | Catalase |
| 461,775254 | 51,08585 | 27,453759 | P12613 | T-complex protein 1 subunit alpha (TCP-1-alpha) |
| 461,492939 | 340,683749 | 202,614909 | A5XCL5 | LD36265p; |
| 459,509076 | 33,470581 | 76,143713 | Q7K3J0 | CG8258; |
| 457,960929 | 177,224872 | 133,310715 | Q9XZT5 | Heatshock protein cognate 70Cb; |
| 454,897288 | 306,494989 | 250,260489 | A9J7N9 | Aldehyde dehydrogenase; |
| 450,558248 | 372,455095 | 426,599213 | Q7KUB0 | Isocitrate dehydrogenase [NADP] |
| 448,713227 | 520,834914 | 305,406962 | A5XD83 | Phosphoglycerate mutase |
| 448,414108 | 258,046023 | 200,263628 | X2JF59 | Thioredoxin peroxidase 1, isoform C; |
| 447,219817 | 175,194149 | 422,930809 | P50887 | 60S ribosomal protein L22 |
| 444,216349 | 146,984646 | 129,353269 | Q9NJH0 | Elongation factor 1-gamma (EF-1-gamma) |
| 433,951305 | 425,885834 | 272,94356 | Q9VNX4 | Delta-1-Pyrroline-5-carboxylate dehydrogenase 1, isoform A; |
| 429,986847 | 582,661581 | 604,392015 | Q9VAC1 | CG7920, isoform A; |
| 419,400371 | 533,662129 | 288,301041 | Q8IGE9 | RH35990p; |
| 416,661093 | 360,4168 | 348,393959 | X2JJG8 | Glutamine synthetase |
| 412,761772 | 237,81562 | 180,525643 | P52034_ISOFORM_C | ATP-dependent 6-phosphofructokinase (ATP-PFK) (Phosphofructokinase) [ISOFORM C] |
| 411,321903 | 180,062836 | 193,844885 | P29310_ISOFORM_VI | 14-3-3 protein zeta [ISOFORM VI] |
| 410,921576 | 366,906389 | 195,429249 | Q8IRQ5 | LP02262p; |
| 410,801167 | 342,888201 | 325,347074 | P29613 | Triosephosphate isomerase (TIM) |
| 408,287645 | 103,482817 | 31,606255 | E2QCF1 | ATP citrate lyase, isoform F; |
| 402,512263 | 197,098553 | 464,522727 | O97118 | Ribosomal protein L23a; |
| 400,889908 | 138,477601 | 87,390673 | P91938_ISOFORM_D | Thioredoxin reductase 1, mitochondrial (TrxR-1) [ISOFORM D] |
| 395,658156 | 154,261196 | 148,273036 | Q9VGZ3 | Aconitate hydratase (Aconitase) |
| 392,454396 | 303,861519 | 399,869156 | Q9V3S0 | Cytochrome P450 4g1 |

| | | | | |
|---|---|---|---|---|
| 386,114722 | 266,751047 | 160,67267 | Q9VSA3_CHAIN_0 | Probable medium-chain specific acyl-CoA dehydrogenase, mitochondrial (MCAD) [CHAIN 0] |
| 385,360793 | 571,062967 | 578,346238 | P22700_ISOFORM_A | Calcium-transporting ATPase sarcoplasmic/endoplasmic reticulum type [ISOFORM A] |
| 384,719985 | 661,831717 | 1404,21046 | Q7K569 | Glycerol-3-phosphate dehydrogenase |
| 382,87125 | 209,263084 | 156,640585 | P29310_ISOFORM_C | 14-3-3 protein zeta [ISOFORM C] |
| 377,988128 | 83,622292 | 36,148282 | Q9VHR8_ISOFORM_2 | Dipeptidyl peptidase 3 (Dipeptidyl arylamidase III) (DPP III) [ISOFORM 2] |
| 376,86154 | 175,965786 | 204,913621 | Q9VL70 | CG4600-PA; |
| 375,526461 | 66,491125 | 85,376067 | Q9V3U6 | 26-29kD-proteinase; |
| 373,057487 | 529,183744 | 262,055072 | Q9VAN7 | GH13304p; |
| 365,865682 | 188,330099 | 209,893887 | O96827_CHAIN_0 | Probable elongation factor 1-beta (EF-1-beta) [CHAIN 0] |
| 365,08187 | 77,557619 | 183,224409 | P02844_CHAIN_0 | Vitellogenin-2 [CHAIN 0] |
| 364,999627 | 306,034142 | 282,749941 | P00334_CHAIN_0 | Alcohol dehydrogenase [CHAIN 0] |
| 363,550243 | 268,02008 | 94,880377 | E2QCS7 | CG17097; |
| 363,468997 | 187,359861 | 118,95484 | Q86BS7 | CG9485, isoform C; |
| 361,551591 | 362,987102 | 160,585902 | F6J7B3 | Peptidyl-prolyl cis-trans isomerase |
| 360,290404 | 132,581076 | 144,119625 | P48602 | V-type proton ATPase catalytic subunit A isoform 1 (V-ATPase subunit A 1) |
| 357,157121 | 40,706526 | 48,227221 | Q0KHS7 | Lipid storage droplet-2, isoform B; |
| 355,474914 | 321,678411 | 108,376337 | P08171_CHAIN_0 | Esterase-6 (Est-6) (Carboxylesterase-6) [CHAIN 0] |
| 353,072476 | 415,228649 | 153,498502 | Q9VM18 | Trehalose 6-phosphate phosphatase |
| 348,339188 | 139,59022 | 102,787523 | Q9V3V6 | 26S proteasome regulatory complex subunit p50; |
| 348,126231 | 83,90927 | 129,382083 | Q9VA91 | 40S ribosomal protein S7 |
| 347,915634 | 162,847141 | 177,236404 | P46150_ISOFORM_C | Moesin/ezrin/radixin homolog 1 (Ezrin-moesin-radixin 1) (dMoesin) [ISOFORM C] |
| 345,554133 | 185,563505 | 422,386765 | P55830_CHAIN_0 | 40S ribosomal protein S3a [CHAIN 0] |
| 342,673578 | 234,736602 | 397,002368 | Q9VFQ9 | Dipeptidase B, isoform A; |
| 342,098764 | 252,286264 | 234,082237 | Q0KHZ6 | CG7834, isoform A; |
| 337,167785 | 176,654781 | 118,95484 | C7LAB1 | SD23689p; |
| 336,224109 | 211,494772 | 284,699096 | Q9W1H8 | GH13256p; |
| 334,783558 | 293,098833 | 271,010535 | Q94523_CHAIN_0 | Succinate dehydrogenase [ubiquinone] flavoprotein subunit, mitochondrial (FP) [CHAIN 0] |
| 327,851055 | 194,958483 | 179,188632 | P82147 | Protein lethal(2)essential for life |
| 327,127602 | 67,475623 | 94,301505 | Q7KMQ0 | 26S proteasome regulatory complex subunit p48B; |
| 324,970759 | 91,74528 | 145,614684 | Q9NIW0 | Malic enzyme |
| 320,890206 | 98,831164 | 65,503248 | A0A0B4KF46 | Ubiquitin activating enzyme 1, isoform C; |
| 320,307968 | 90,771667 | 60,156221 | Q9W227 | Peptidyl-prolyl cis-trans isomerase |
| 315,935926 | 407,625745 | 234,815405 | Q7K2P3 | CG1648, isoform A; |
| 314,267241 | 143,548773 | 182,151716 | Q9GU68 | Eukaryotic translation initiation factor 5A (eIF-5A) |
| 311,344371 | 257,898529 | 147,95719 | A1Z992 | 1,4-Alpha-Glucan branching enzyme; |
| 305,33606 | 141,515313 | 157,378632 | Q8T0M9 | Aspartate aminotransferase |

| | | | | |
|---|---|---|---|---|
| 300,633722 | 30,643867 | 26,048889 | Q9VL18 | Probable elongation factor 1-delta (EF-1-delta) |
| 300,236688 | 313,892332 | 252,292395 | A0A077HCQ5 | Alcohol dehydrogenase |
| 299,89885 | 76,47389 | 54,983017 | Q7YWB4 | Aminotransferase; |
| 295,613123 | 197,663046 | 156,667671 | B7Z0X1 | Serine hydroxymethyltransferase |
| 291,417687 | 144,30914 | 139,566179 | A0A0B4LJ12 | Nucleoside diphosphate kinase |
| 289,918305 | 128,157938 | 47,87112 | Q9VPX7 | Adenylyl cyclase-associated protein |
| 288,405645 | 54,568304 | 67,130565 | P06606 | Tubulin alpha-4 chain |
| 285,877749 | 46,195988 | 23,835301 | O18413 | 26S protease regulatory subunit 8 |
| 285,588357 | 172,038383 | 93,591824 | Q9TWZ1_CHAIN_0 | Protein disulfide-isomerase [CHAIN 0] |
| 284,931699 | 258,896918 | 147,936404 | Q9VTK9 | CG6084, isoform A; |
| 283,566335 | 172,038383 | 85,734519 | Q3YMU0_CHAIN_0 | Protein disulfide-isomerase [CHAIN 0] |
| 282,263141 | 84,798696 | 25,835729 | Q9VAY2 | Glycoprotein 93; |
| 279,434659 | 109,835163 | 94,021805 | Q9XZC3 | Tat-binding protein-1; |
| 277,169701 | 410,398371 | 103,930631 | Q9VFC8_ISOFORM_B | Glycogen [starch] synthase [ISOFORM B] |
| 276,811032 | 72,47457 | 282,773644 | X2JIQ5 | Ribosomal protein L17, isoform E; |
| 276,191395 | 243,989913 | 301,209383 | P61851_CHAIN_0 | Superoxide dismutase [Cu-Zn] [CHAIN 0] |
| 276,157147 | 104,58745 | 124,75633 | Q7KN94 | Walrus, isoform A; |
| 275,086409 | 128,674623 | 87,015861 | Q9VKW5 | CG5355; |
| 274,52847 | 96,329454 | 251,958561 | P19889 | 60S acidic ribosomal protein P0 |
| 273,006399 | 135,249258 | 105,944508 | Q9VUY9 | Phosphoglucomutase (PGM) |
| 271,876446 | 54,101802 | 40,202736 | P48601 | 26S protease regulatory subunit 4 (P26s4) |
| 268,880719 | 73,134722 | 133,317543 | P80455 | 40S ribosomal protein S12 |
| 265,201003 | 141,302052 | 121,96711 | P45594 | Cofilin/actin-depolymerizing factor homolog |
| 263,267754 | 51,99258 | 154,572462 | Q9VFV9 | DnaJ-like-2, isoform A; |
| 263,022885 | 103,843547 | 118,889753 | P41043 | Glutathione S-transferase S1 |
| 262,651327 | 67,863472 | 43,219563 | Q24150 | Nucleosome assembly protein NAP-1; |
| 261,888366 | 234,229552 | 79,102421 | C8VV33 | AT04852p; |
| 252,192791 | 146,964201 | 262,295251 | P39018_CHAIN_0 | 40S ribosomal protein S19a [CHAIN 0] |
| 248,970738 | 437,439112 | 230,403015 | Q7K2W6 | GH04080p; |
| 248,499425 | 146,11769 | 323,48709 | P41126 | 60S ribosomal protein L13 |
| 248,240647 | 156,066696 | 75,49719 | Q9VKI8 | CG6287-PA; |
| 244,779716 | 154,636223 | 222,734831 | Q9VWG3 | 40S ribosomal protein S10b |
| 243,596917 | 150,874607 | 105,65039 | Q8T9D5 | SD05601p; |
| 242,99753 | 85,182745 | 71,837652 | Q9VY05 | CG9512, isoform A; |
| 239,70013 | 216,919743 | 216,98886 | Q95U46 | GH07925p; |
| 238,886635 | 139,605786 | 196,742536 | P46223 | 60S ribosomal protein L7a |
| 237,079278 | 61,091491 | 223,294051 | Q9V9W2 | Ribosomal protein L6, isoform A; |
| 236,329066 | 61,144176 | 47,595579 | Q9V3G7 | 26S proteasome non-ATPase regulatory subunit 6 |
| 231,846937 | 63,612444 | 102,951082 | Q9VN25 | Eukaryotic translation initiation factor 3 subunit A (eIF3a) |
| 231,697932 | 162,696678 | 246,078838 | Q9V3G1 | 60S ribosomal protein L8 |
| 230,301466 | 83,09705 | 80,206226 | O18640 | Guanine nucleotide-binding protein subunit beta-like protein |

| | | | | |
|---|---|---|---|---|
| 229,619243 | 349,423957 | 56,782575 | A8DZ29 | Eukaryotic translation initiation factor 4G, isoform B; |
| 223,670132 | 163,021009 | 205,649765 | Q8MLY8 | 40S ribosomal protein S8 |
| 223,474079 | 37,909018 | 123,381293 | O02195 | Eukaryotic translation initiation factor 3 subunit I (eIF3i) |
| 223,321266 | 148,346493 | 74,78204 | Q4V3L7 | IP10537p; |
| 222,61468 | 135,05619 | 117,60346 | O16043 | Anon1A4; |
| 221,678258 | 102,777342 | 72,545258 | P29413_CHAIN_0 | Calreticulin [CHAIN 0] |
| 221,417859 | 116,916527 | 287,972811 | Q24208 | Eukaryotic translation initiation factor 2 subunit 3 (eIF-2-gamma) |
| 220,368593 | 166,83438 | 129,03681 | Q960M4 | LD45324p; |
| 219,032305 | 160,831577 | 87,181973 | E1JGZ9 | Proteasome subunit alpha type |
| 217,48371 | 118,382191 | 71,536697 | Q95U34 | GH11113p; |
| 216,452366 | 906,30065 | 39,326413 | M9PF14 | Trailer hitch, isoform C; |
| 215,796774 | 127,775587 | 95,343355 | Q9V521 | Phenoloxidase 2 (PO A3) (Pro-phenoloxidase A3) |
| 215,700383 | 139,379468 | 70,292219 | O97428 | CG4944-PA, isoform A; |
| 214,886508 | 37,901043 | 323,801622 | O16797_CHAIN_0 | 60S ribosomal protein L3 [CHAIN 0] |
| 212,722271 | 142,762307 | 146,316781 | A0A0B4LGS4 | Vacuolar H[+]-ATPase 26kD subunit, isoform C; |
| 210,767097 | 77,084631 | 63,008036 | Q9VLB7 | GDP dissociation inhibitor, isoform A; |
| 208,225792 | 21,991848 | 130,476299 | O61231 | 60S ribosomal protein L10 |
| 205,544904 | 124,138905 | 63,190032 | P46415 | Alcohol dehydrogenase class-3 (FALDH) (FDH) (GSH-FDH) |
| 203,716305 | 171,125212 | 77,74765 | Q8SY61_CHAIN_0 | General odorant-binding protein 56d [CHAIN 0] |
| 203,586541 | 328,613844 | 220,055792 | Q9VTC3 | CG6409, isoform B; |
| 202,311477 | 70,533643 | 108,397735 | O96553_ISOFORM_A | C-1-tetrahydrofolate synthase, cytoplasmic (C1-THF synthase) [ISOFORM A] |
| 202,013312 | 182,143475 | 118,397462 | Q9VGM2 | Fatty acid bindin protein, isoform B; |
| 199,308416 | 72,046176 | 287,11272 | Q5U0W4 | GM06787p; |
| 199,217577 | 125,989053 | 231,718388 | Q06559 | 40S ribosomal protein S3 |
| 198,070351 | 1031,026964 | 36,712747 | P21187 | Polyadenylate-binding protein (PABP) (Poly(A)-binding protein) |
| 197,891404 | 70,632436 | 72,562805 | P14318 | Muscle-specific protein 20 |
| 191,668283 | 55,454529 | 41,429208 | Q7JR58 | CG6543, isoform A; |
| 188,879456 | 69,229343 | 42,714193 | P22769 | Proteasome subunit alpha type-7-1 |
| 187,262296 | 209,069945 | 83,626791 | Q9VXI1 | CG9914, isoform A; |
| 185,503778 | 155,13832 | 188,016626 | Q9VEN1_ISOFORM_2 | Filamin-A (FLN-A) (ABP-280) [ISOFORM 2] |
| 185,375775 | 58,01788 | 64,945576 | A0A0B4JD95 | Zipper, isoform H; |
| 185,119879 | 86,773955 | 138,150946 | Q9VJY6 | 60S ribosomal protein L24 |
| 184,518696 | 34,457488 | 203,357118 | P41092 | 60S ribosomal protein L27a |
| 184,515685 | 86,368691 | 36,170898 | M9PE40 | Ubiquitin carboxyl-terminal hydrolase |
| 182,511183 | 33,246042 | 123,102052 | P36241 | 60S ribosomal protein L19 |
| 182,07736 | 165,393698 | 181,922912 | P29746 | Protein bangles and beads |
| 181,886725 | 107,699861 | 34,284156 | Q8IPF5 | A kinase anchor protein 200, isoform B; |
| 179,404829 | 81,426256 | 162,137258 | Q9VA83 | Ferritin |
| 178,928196 | 143,633478 | 182,302059 | P41042 | 40S ribosomal protein S4 |

| | | | | |
|---|---|---|---|---|
| 178,689483 | 224,1845 | 315,557749 | P48604 | GrpE protein homolog, mitochondrial |
| 178,675959 | 215,848124 | 171,705591 | P29843 | Heat shock 70 kDa protein cognate 1 |
| 178,233795 | 29,978522 | 318,406048 | Q9VKC3 | CG14945, isoform B; |
| 175,403322 | 118,259718 | 185,079211 | Q9W2X6 | Lethal (1) G0230, isoform A; |
| 174,636051 | 116,387206 | 45,070103 | X2JES1 | Fructose-1,6-bisphosphatase, isoform C; |
| 174,183099 | 100,605166 | 56,953425 | Q9VW68 | CG7433, isoform A; |
| 174,002324 | 112,590414 | 177,38828 | Q9VNB9 | RE17737p; |
| 173,597877 | 260,678065 | 92,04987 | P54192_CHAIN_0 | General odorant-binding protein 19d (PBPRP-2) [CHAIN 0] |
| 173,035459 | 50,507001 | 48,770159 | F3YDF6 | MIP08376p; |
| 172,4674 | 78,337738 | 34,284156 | Q86BM5 | A kinase anchor protein 200, isoform G; |
| 172,227266 | 85,022888 | 112,665841 | Q9V5C6 | Proteasome subunit alpha type-3 |
| 170,064487 | 127,303691 | 174,782717 | Q00637_CHAIN_0 | Superoxide dismutase [Mn], mitochondrial [CHAIN 0] |
| 170,032431 | 82,804143 | 116,37461 | P29327 | 40S ribosomal protein S6 |
| 169,350332 | 170,444772 | 164,250637 | Q9VG58 | Major heat shock 70 kDa protein Bbb (Heat shock protein 70Bbb) |
| 167,518232 | 67,409689 | 21,773503 | Q9VZ23 | GTP-binding nuclear protein Ran |
| 165,548247 | 255,391852 | 166,913428 | Q9GV29 | Prophenoloxidase; |
| 164,632246 | 115,359228 | 214,941778 | Q9VHE5 | RH48056p; |
| 164,462878 | 100,090837 | 68,216371 | P18053 | Proteasome subunit alpha type-4 |
| 163,227322 | 39,24787 | 35,437112 | Q9VJJ0 | Proteasome subunit beta type |
| 162,16335 | 45,273093 | 123,103935 | P48159 | 60S ribosomal protein L23 |
| 161,605363 | 850,968145 | 39,326413 | Q9VTZ0 | GH08269p; |
| 160,014023 | 79,379395 | 86,560143 | Q9VTV3 | AT17975p; |
| 159,35522 | 95,871533 | 98,6337 | P42281 | Acyl-CoA-binding protein homolog (ACBP) (DBI) |
| 159,167688 | 46,976243 | 38,240976 | Q9VRP2 | CG10576, isoform A; |
| 158,607796 | 99,25461 | 126,3997 | P31009 | 40S ribosomal protein S2 |
| 157,396033 | 160,04301 | 231,399013 | Q24251 | ATP synthase subunit d, mitochondrial (ATPase subunit d) |
| 155,603356 | 75,423693 | 35,503888 | Q9VXF1 | Serine/threonine-protein phosphatase 2B catalytic subunit 3 |
| 155,274736 | 204,834245 | 91,185513 | Q9VBP6 | GH21316p; |
| 155,22557 | 177,639934 | 117,533042 | Q9VWG1 | CG14207, isoform D; |
| 153,669583 | 345,309674 | 223,767754 | Q8T8R1 | CCHC-type zinc finger protein CG3800 |
| 152,120741 | 217,102881 | 175,549612 | Q29QY4 | Alcohol dehydrogenase |
| 149,849866 | 94,419347 | 213,445817 | R9Q794 | Ribosomal protein L5, isoform G; |
| 148,453758 | 84,161851 | 39,916132 | Q9VU68 | Actin-interacting protein 1 (AIP1) |
| 148,056343 | 174,077103 | 117,075586 | P20432 | Glutathione S-transferase 1-1 |
| 146,137809 | 65,692669 | 139,500494 | Q9W237 | 40S ribosomal protein S16 |
| 145,999276 | 174,48867 | 176,418364 | O97125 | Heat shock protein 68 |
| 145,611952 | 58,17417 | 192,934029 | P41094 | 40S ribosomal protein S18 |
| 145,138384 | 115,145227 | 114,456216 | Q9VZQ8 | Glutathione peroxidase |
| 140,430146 | 169,401605 | 270,792802 | O01666 | ATP synthase subunit gamma, mitochondrial |
| 139,086242 | 34,811645 | 94,179593 | X2JFR6 | Eukaryotic translation initiation factor 2alpha, isoform B; |

| | | | | |
|---|---|---|---|---|
| 138,456968 | 25,289949 | 53,986135 | P20477_CHAIN_0 | Glutamine synthetase 1, mitochondrial [CHAIN 0] |
| 138,1305 | 32,854311 | 96,977125 | Q9U1J0 | Malic enzyme |
| 137,562161 | 88,445092 | 106,04889 | P48588 | 40S ribosomal protein S25 |
| 137,383057 | 33,969185 | 159,988119 | X2JDU0 | Ribosomal protein L9, isoform C; |
| 135,710616 | 63,702258 | 162,56849 | Q9VBH8 | FI02850p; |
| 135,514329 | 82,565146 | 125,252409 | Q9V9M7 | RE62581p; |
| 134,576259 | 78,348455 | 58,358699 | P41093 | 60S ribosomal protein L18a |
| 133,688734 | 87,811473 | 70,209556 | Q9U915 | Adenylate kinase |
| 133,681824 | 48,102411 | 58,01738 | Q7K485 | CathD; |
| 133,412896 | 126,868494 | 85,516972 | Q95RI5 | Failed axon connections |
| 133,189864 | 103,062091 | 249,215617 | O17445 | 60S ribosomal protein L15 |
| 132,627402 | 49,024923 | 15,63152 | M9PGX2 | Pretaporter, isoform D; |
| 131,886851 | 96,232075 | 47,767708 | P23779 | Cystatin-like protein |
| 131,719408 | 79,250374 | 100,956592 | Q7KT11 | CG9331, isoform B; |
| 131,703256 | 72,076308 | 77,064957 | D1FZD8 | Odorant-binding protein 99c; |
| 131,665841 | 83,695534 | 81,515306 | Q9VFF0 | CG3731, isoform A; |
| 131,583306 | 118,505402 | 225,49882 | Q7JV23 | Acetyl-CoA carboxylase, isoform B; |
| 129,930618 | 61,782779 | 23,520981 | Q9I7S8 | Multifunctional protein ADE2 |
| 129,226124 | 61,207145 | 55,072738 | Q7KMR7 | Thioredoxin-like protein TXL; |
| 127,442176 | 250,885952 | 134,695209 | Q7K084 | Odorant-binding protein 44a, isoform A; |
| 125,463207 | 75,77324 | 103,32149 | P84029 | Cytochrome c-2 |
| 122,456676 | 88,564566 | 105,899743 | A0A0B4LFD9 | Ribosomal protein S23, isoform B; |
| 121,922952 | 77,73343 | 89,573043 | O76927_ISOFORM_E | 40S ribosomal protein S21 [ISOFORM E] |
| 120,724349 | 78,7372 | 208,418505 | Q9VBN5 | 60S ribosomal protein L27 |
| 119,955099 | 87,989397 | 38,030803 | Q9VKX2 | Malate dehydrogenase |
| 119,186118 | 31,093236 | 188,383563 | M9PHM6 | 60S ribosomal protein L18 |
| 119,112119 | 238,340751 | 93,838339 | E1JHG2 | Seminal fluid protein 33A3; |
| 116,637747 | 67,278155 | 28,546481 | Q9W0E4 | Puromycin sensitive aminopeptidase, isoform A; |
| 116,406575 | 37,510775 | 52,981412 | Q9W4X7 | Eukaryotic translation initiation factor 3 subunit G-1 (eIF3g-1) (eIF-3 RNA-binding subunit 1) |
| 115,915908 | 87,61503 | 46,546594 | Q9VLR5 | RNA polymerase II transcriptional coactivator |
| 115,526103 | 119,50817 | 117,32896 | Q9VU35 | CG11267-PA; |
| 115,344294 | 98,931839 | 160,825895 | Q9V8Y2_CHAIN_0 | General odorant-binding protein 56a [CHAIN 0] |
| 114,95126 | 77,224104 | 48,865698 | Q9VXK0 | Protein NipSnap |
| 114,614346 | 66,11217 | 69,158644 | Q9W334 | 40S ribosomal protein S28 |
| 114,562105 | 54,7434 | 47,930948 | P35554 | Flightin |
| 114,251321 | 17,489347 | 15,956593 | Q9VSL4 | Glutathione S transferase O2, isoform B; |
| 112,911351 | 85,919238 | 128,722075 | Q7JYK1 | RE10554p; |
| 111,420271 | 111,329177 | 160,113166 | Q24439 | ATP synthase subunit O, mitochondrial (OSCP) |
| 110,834505 | 153,19269 | 114,87988 | Q9W1C9_CHAIN_0 | Ejaculatory bulb-specific protein 3 [CHAIN 0] |
| 110,788116 | 99,595142 | 88,646394 | P02516 | Heat shock protein 23 |

| | | | | |
|---|---|---|---|---|
| 110,654678 | 87,489418 | 131,922169 | Q8IGR0 | RE44350p; |
| 110,550864 | 17,254082 | 38,302749 | Q9VGS2 | Translationally-controlled tumor protein homolog (TCTP) |
| 110,41305 | 69,648872 | 49,147198 | A0APE4 | CG6459 protein; |
| 110,383961 | 125,976052 | 21,501629 | Q9VFT4 | AT27578p; |
| 109,461387 | 96,110725 | 83,581975 | Q9VSP9 | CG5804-PA; |
| 108,510511 | 46,282571 | 119,403327 | Q03334 | 40S ribosomal protein S13 |
| 107,246968 | 60,35119 | 130,05053 | X2JCX8 | Ribosomal protein S14b, isoform B; |
| 106,877199 | 102,614067 | 43,040377 | P22465 | Annexin B10 |
| 106,034367 | 34,739395 | 147,475177 | X2JC35 | 60S ribosomal protein L36 |
| 105,012154 | 71,465722 | 93,040992 | O97479 | FI05212p; |
| 104,280421 | 41,442518 | 49,819076 | Q9VCR2 | Aminoacylase-1 |
| 103,423238 | 14,694978 | 114,049852 | Q9VLT7 | CG7424-PA; |
| 102,760644 | 120,51854 | 120,72489 | Q9Y125 | BcDNA.GH08312; |
| 101,556719 | 109,721192 | 22,542724 | Q9W2J5 | CG44245, isoform A; |
| 101,345299 | 58,841662 | 85,197082 | Q7JX87 | Peroxiredoxin 2540-2; |
| 100,987639 | 114,48412 | 118,847063 | Q9VL68 | CG4598, isoform B; |
| 100,074213 | 47,337077 | 60,468352 | O62621 | Coatomer subunit beta' (Beta'-COP) |
| 99,635425 | 77,744088 | 68,53998 | A0A0B4JD48 | Cysteine proteinase-1, isoform D; |
| 99,409876 | 15,991021 | 121,902165 | Q9VNE9 | 60S ribosomal protein L13a |
| 97,806001 | 86,416489 | 51,881191 | Q9VQB4 | CG3609, isoform B; |
| 97,069796 | 36,279598 | 108,080647 | Q9VAI9 | Odorant-binding protein 99c, isoform A; |
| 96,406823 | 84,290414 | 205,439244 | X2J5G6 | Ribosomal protein L7, isoform B; |
| 96,26933 | 75,325672 | 15,527324 | Q29QY5 | IP15841p; |
| 95,991168 | 131,286837 | 326,040739 | Q868Z9_CHAIN_0 | Papilin [CHAIN 0] |
| 94,181141 | 91,728579 | 49,69256 | P62152_CHAIN_0 | Calmodulin (CaM) [CHAIN 0] |
| 92,999281 | 119,65166 | 104,593953 | Q9VJ44 | CG17597, isoform B; |
| 92,376488 | 55,20429 | 42,989228 | Q7KW39_ISOFORM_A | Probable methylmalonate-semialdehyde dehydrogenase [acylating], mitochondrial (MMSDH) (Malonate-semialdehyde dehydrogenase [acylating]) [ISOFORM A] |
| 92,089093 | 60,619131 | 232,017511 | Q7JWF1 | Electron transfer flavoprotein-ubiquinone oxidoreductase, isoform A; |
| 91,690135 | 51,428968 | 34,569617 | O97102 | Small ubiquitin-related modifier (SUMO) |
| 91,22479 | 87,631386 | 42,975727 | P20228 | Glutamate decarboxylase (GAD) |
| 90,649248 | 92,453844 | 64,312226 | Q7KRU8 | Ferritin |
| 89,524318 | 42,575857 | 35,06719 | Q9XZH6 | V-type proton ATPase subunit G (V-ATPase subunit G) |
| 88,925822 | 37,856587 | 126,471807 | Q24407 | ATP synthase-coupling factor 6, mitochondrial (ATPase subunit F6) |
| 88,24906 | 37,052433 | 100,03847 | Q9U9Q7 | Transcription factor BTF3 |
| 86,098413 | 58,841662 | 68,29978 | A1Z893 | CG12896; |
| 85,354776 | 216,258276 | 188,562807 | P09491 | Tropomyosin-2 |
| 85,180688 | 131,766794 | 76,383532 | Q6NN12 | HL05792p; |
| 84,644763 | 120,45756 | 118,933485 | Q9W596 | Futsch light chain LC(f) |
| 84,506998 | 211,269591 | 162,478873 | Q26365_ISOFORM_A | ADP,ATP carrier protein (ANT) [ISOFORM A] |

| | | | | |
|---|---|---|---|---|
| 84,02598 | 80,321279 | 27,409917 | Q6NL47 | HL05814p; |
| 82,793068 | 60,980793 | 173,004507 | L0CRF4 | Ribosomal protein S11; |
| 82,702144 | 63,745375 | 68,274115 | Q94518 | Nascent polypeptide-associated complex subunit alpha (NAC-alpha) |
| 82,481279 | 98,220794 | 43,930289 | Q9V9S8 | Ferrochelatase, mitochondrial |
| 81,308525 | 95,437534 | 98,967992 | P41964_CHAIN_0 | Drosomycin [CHAIN 0] |
| 81,305383 | 128,270487 | 164,883868 | P21914 | Succinate dehydrogenase [ubiquinone] iron-sulfur subunit, mitochondrial (Ip) |
| 81,066951 | 96,937625 | 131,785489 | Q27869 | Protein-L-isoaspartate(D-aspartate) O-methyltransferase (PIMT) |
| 80,496723 | 181,31668 | 231,499119 | Q06943 | High mobility group protein Z (HMG-Z) |
| 79,928089 | 86,229328 | 124,704784 | Q9VV75 | AT02348p; |
| 79,760708 | 47,612203 | 30,408657 | Q9V6U9_CHAIN_0 | Probable trans-2-enoyl-CoA reductase, mitochondrial [CHAIN 0] |
| 79,518528 | 77,668252 | 114,656929 | Q9VZS5 | 60S ribosomal protein L28 |
| 74,524538 | 162,066606 | 154,080007 | P06754_ISOFORM_D | Tropomyosin-1, isoforms 9A/A/B [ISOFORM D] |
| 73,521429 | 53,075553 | 36,444153 | Q8T3W8 | AT27079p; |
| 72,53784 | 22,649181 | 92,383613 | Q9VJ28 | CG10639, isoform A; |
| 69,853536 | 145,347953 | 124,671538 | Q058U3 | IP16008p; |
| 69,557749 | 34,552175 | 76,034647 | Q8SYS2 | RE38869p; |
| 69,45322 | 96,630751 | 122,056972 | Q9V426 | LD07162p; |
| 69,129371 | 68,601536 | 58,717393 | O77134 | 9 kD basic protein; |
| 68,52543 | 74,29161 | 20,119801 | Q8IR95 | RH08789p; |
| 68,237555 | 65,978042 | 62,599217 | Q9VN93_CHAIN_0 | Putative cysteine proteinase CG12163 [CHAIN 0] |
| 67,1989 | 65,056739 | 79,623521 | Q9VD01 | LP12095p; |
| 66,91566 | 100,379339 | 76,434219 | Q9U982 | Drosophila dodeca-satellite protein 1; |
| 66,024944 | 20,026843 | 38,29339 | Q07171_ISOFORM_2 | Gelsolin [ISOFORM 2] |
| 64,572815 | 92,177733 | 155,173936 | P53777 | Muscle LIM protein 1 |
| 62,670544 | 45,077529 | 70,540288 | E1JJM9 | Ribosomal protein S15Aa, isoform F; |
| 62,403852 | 52,971413 | 29,351081 | P35421 | Phosphoribosylformylglycinamidine synthase (FGAM synthase) (FGAMS) (FGAR amidotransferase) (FGAR-AT) |
| 61,88491 | 48,59762 | 39,658638 | Q9VK99 | Atilla, isoform B; |
| 61,775846 | 46,93396 | 337,908557 | Q24388_CHAIN_0 | Larval serum protein 2 (LSP-2) [CHAIN 0] |
| 61,55963 | 81,720072 | 60,269769 | Q9VY92 | CG11151, isoform B; |
| 61,421159 | 103,304491 | 87,700409 | Q9XZS3 | Translation machinery-associated protein 7 homolog |
| 59,95915 | 78,424329 | 36,73261 | O46200_CHAIN_0 | Accessory gland protein Acp63F [CHAIN 0] |
| 59,108801 | 43,111811 | 65,899578 | Q9V597 | 60S ribosomal protein L31 |
| 58,132213 | 16,609637 | 28,787244 | Q9W0J9 | CG9119; |
| 57,106468 | 49,063286 | 38,086708 | Q9VTI5 | CG6140, isoform A; |
| 56,427971 | 54,964826 | 43,541736 | Q9VQF7 | Bacchus, isoform B; |
| 54,868937 | 52,473835 | 60,952488 | P02283_CHAIN_0 | Histone H2B [CHAIN 0] |
| 53,958156 | 52,378202 | 17,502292 | M9PB52 | Galactose-1-phosphate uridylyltransferase |
| 51,503927 | 191,896383 | 360,234385 | Q94920_CHAIN_0 | Voltage-dependent anion-selective channel [CHAIN 0] |

| | | | | |
|---|---|---|---|---|
| 48,371219 | 162,832547 | 79,510373 | Q9VMV9 | Reticulon-like protein |
| 47,438138 | 51,075777 | 41,540479 | Q8IPM8_ISOFORM_C | Complexin [ISOFORM C] |
| 44,4725 | 81,540221 | 187,202276 | Q94516 | ATP synthase subunit b, mitochondrial (ATPase subunit b) |
| 42,685694 | 64,476362 | 61,100165 | Q9W4V3 | CG14419-PA; |
| 41,14575 | 397,305359 | 49,13281 | Q08473_ISOFORM_A | RNA-binding protein squid (HNRNP 40) [ISOFORM A] |
| 41,121509 | 73,998559 | 134,174574 | Q24201 | Carboxylic ester hydrolase |
| 40,378801 | 50,960744 | 77,205122 | Q9VD29 | RE74312p; |
| 39,515698 | 43,857904 | 37,661499 | Q8SYQ8 | CG3420, isoform A; |
| 38,541423 | 40,358137 | 42,325594 | Q9V7N5_ISOFORM_A | V-type proton ATPase subunit C (V-ATPase subunit C) [ISOFORM A] |
| 37,841728 | 101,170828 | 220,395279 | P13607_ISOFORM_3 | Sodium/potassium-transporting ATPase subunit alpha (Na(+)/K(+) ATPase alpha subunit) [ISOFORM 3] |
| 37,709448 | 22,91279 | 29,58324 | Q0E940 | Eukaryotic translation initiation factor 3 subunit B (eIF3b) |
| 34,99479 | 15,921154 | 61,647788 | Q9W229 | 40S ribosomal protein S24 |
| 34,634988 | 62,116106 | 14,750294 | X2JFD6 | Peroxisomal multifunctional enzyme type 2, isoform B; |
| 34,415161 | 50,439013 | 31,632846 | Q94519_ISOFORM_1 | Acyl carrier protein, mitochondrial (ACP) [ISOFORM 1] |
| 32,964941 | 122,835312 | 128,601102 | Q8IQW2 | CG14235-PB, isoform B; |
| 32,819429 | 110,918178 | 27,800506 | P41073_ISOFORM_C | Zinc finger protein on ecdysone puffs [ISOFORM C] |
| 27,839934 | 22,570223 | 111,21573 | X2JB24 | Neuropeptide-like 2, isoform B; |
| 26,377039 | 51,869708 | 57,908242 | Q9VY87 | Cathepsin B1, isoform A; |
| 26,08523 | 21,653926 | 38,198019 | Q9VZI1 | Transgelin |
| 25,072566 | 52,523143 | 35,624803 | Q9W306 | CG9691, isoform B; |
| 18,19336 | 49,998171 | 37,145852 | M9PFX9 | Limpet, isoform N; |
