# Supplementary material for: Mms19 promotes spindle microtubule assembly in Drosophila neural stem cells
Source: PLoS Genet. 2020 Nov 19;16(11):e1008913. doi: 10.1371/journal.pgen.1008913 (PMC7714366; doi:10.1371/journal.pgen.1008913)
Supplement: Attachment — Submitted filename: FINAL Reviewers Comments.pdf [file pgen.1008913.s018.pdf]

Response to Reviewers' comments

Reviewer #1:

In this manuscript Chippalkatti and co-workers present a thorough analysis of the Mms19 protein and its function in regulating microtubule polymerisation using neural stem cells of the developing fly brain as a model system. Using genetics, quantitative live cell imaging, immunoprecipitation and mass spectrometry analysis as well as Tubulin binding assays the authors report that Mms19 plays a dual role in regulating the morphology of the mitotic spindle. Mms19 is known to be required for nucleotide excision repair. The new roles uncovered here, suggest that Mms19 also plays an important role in microtubule regulation. The study finds that mms19 mutants have smaller brains due to malformation of the optic lobes of the brains. The study then goes on to measure the cellular phenotypes focusing on microtubules of neuroblasts in the central brain. Using these cells, the study reports that one role of MMs19 is to stimulate microtubule nucleation from the centrosome by controlling the recruitment of TACC/Msps, providing novel regulatory insights into centrosomal regulation. In addition, the study reports that Mms19 directly binds to microtubules and is likely to regulate microtubule stability, which could explain the observed phenotypes. The rationale is well explained, and the results are well written. The experimental design appears to be sound and the interpretations supported by experimental evidence.
So, it appears that Mms19 regulates mitotic microtubules as well as postmitotic microtubules. This slows down mitosis in rapidly dividing cells causing phenotypes and is likely due to a role in reinforcing robustness and stability in the mitotic spindle apparatus. To me this makes sense. The findings are also of general interest to a readership such as that of PLOS Genetics and I would in principle support publication, but the authors may wish to look a few points that I listed below.
The manuscript needs some work on figures and their legends in terms of error bars, scale bars and would benefit from some further quantification (see below). I would also try to avoid reporting results in the discussion (e.g. line 395 onwards). The discussion could be improved, working out better the part on the interpretation of how Mms19 works in the cell biological level and another to discuss the broader relevance. Perhaps not all detail present right now is really necessary (I appreciate all the work that has gone into this study, but the ubiquitination part is perhaps not necessary here, I would think that the MS results are also not necessary either, as the results have not really been followed up, they are definitively interesting though).

Comments:
Major:
The figure legends could be improved by clearly stating the number of independent biological repeats in all cases. Some of the graphs also miss error and scale bars and some essential statistical tests and further quantifications are missing, that might be important to include.

➔ *We have revised the figure legends and indicated the number of independent biological replicates for each experiment and also the exact sample size for each genotype in the figure legends. Other, soecific additions are outlined in more detail below.*

Other:
Fig 1: mms19 brains are smaller, NB number the same. How is that? Explanation: mitosis takes longer (mitotic index EdU + live imaging data) and logically MARCM clones produce less offspring. Rescued by Mms19:eGFP, while overexpression of CAK does not. Convincing Comment: A lot of mitosis occurs in the neuroepithelium in developing brains. Da>CAK, Mms19p (Fig 1A) is the OL more damaged. i.e. accelerated cell cycle harmful in this context to this tissue or unlucky picture? So, the brain morphology appears to be largely damaged by faulty neuroepithelial processes, perhaps mentioned that in the manuscript?
➔ *We do not think that the optic lobe is more damaged due to accelerated cell cycle. In the image that we had used in Fig 1A,B, some sections of the brain were perhaps not stained properly or mechanically damaged, which could be the reason why the OL appeared to be damaged. In most of the other images the OL appears smaller but without any other morphological defects. We have replaced the image in the Fig 1A,B.*

Fig 1D is only referring to NBs in the "red shaded" area I guess, if so clarify.
➔ *Thanks for pointing this out. In the graph in Fig 1D (now 1C), we have quantified NBs in the red shaded area which outlines the central brain region. We are stating this now appropriately in the figure legend.*

Line 917: Ph3 labels also dividing GMCs as well as neuroepitheial cells in the OL

To clarify this, we have rephrased this part as follows:
3rd instar larval brain NBs were visualized by staining for Miranda (red, cytoplasmic). They were also stained for pH3 (white, nuclear) and DNA (blue, Hoechst 33342 dye).

Fig 2+3+4
Spindle assembly is driven by centrosomal microtubule nucleation, but also by centrosome independent microtubule nucleation pathways. For instance, cells without centrioles form spindles, yet lack astral microtubules. It is a bit of a shame that this has not been looked into in more detail. Do the authors think that Mms19 plays a role in stabilizing a microtubules or just one pool (astral versus main spindle?)

➔ *Our interpretation of the data is that Mms19 aids the assembly of microtubules (MT) from centrosomes as well as from around the chromatin but we see a more prominent defect in MT assembly from centrosomes in the $Mms19^P$ background. This interpretation is supported by data in the updated S5A-C Fig and movies 03 and 08. In S5A-C Fig at t=3min, we can see that MT nucleation from one spindle pole was delayed while MTs emanate from the other spindle pole as well as from around the chromatin region. We see a dense MT signals in the region between the two poles that approximately corresponds to area around chromatin). In S5A-C Fig, we also clearly see that spindle MT assembly from one pole is delayed. Interestingly, the centrosome which shows a delay in spindle assembly is inherited by the GMC in both cases (Fig S5A-C Fig). Centrosome asymmetry in NBs has been described before (Januschke et al, 2011) and it is known that the GMC-fated centrosome lacks an aster during interphase but assembles microtubules just before NEBD onset. We also observe this MT nucleation delay from one pole in other $Mms19^P$ NBs, but this phenomenon is more striking in the presented examples in S5A-C Fig. Further, the fact that the spindle slightly rotates (shows orientation defects) suggests that astral MT function is clearly affected. Additionally, in Fig 3G-H, we quantified fluorescent intensity of astral as well as the inner spindle MTs and we find a significant decrease in the MT intensity in the astral and the inner spindles in the $Mms19^P$ NBs. Because the chromatin-nucleated MTs also contribute to the inner spindle MT mass, the decrease in the inner spindle density could also be caused by problems in the assembly of chromatin-nucleated MTs. In summary, we found evidence that both types require Mms19 and we now present this evidence better in the new version.*

Fig 3G, what exactly is quantified here is unclear. It reads like this is used to measure spindle length versus cell diameter to quantify spindle length. Provide an example of a fixation of astral microtubules in the mutant. ---
==> In the updated manuscript, this is now Figure 3 E, F. We have now explained the quantification method for measuring relative astral MT density in the figure legend. We also added an image of a fixed $Mms19^P$ cell to show defects in astral MT density in $Mms19^P$ and one showing a very short spindle.

Fig 3 B': the signal is hard to detect. Perhaps a more accurate way would have been to look at the EB1 data in live? Less EB1 comets from centrosomes? ---
➔ *The signal of MTs in general is lower in $Mms19^P$ cells. The lower signal of astral MTs in the $Mms19^P$ cells seems to reflect reduced stability. We tried to measure the number of astral MTs emanating from the centrosomes but did not see a drastic difference, possibly indicating that initiation is normal. However, as the MT plus tip velocity is decreased in the $Mms19^P$ NBs (Fig 4C), we think that this reflects problems in establishing a robust astral MT network and that this leads to a decreased density in fixed NBs.*

Do spindles first form over chromatin and the centrosomal microtubule nucleation is slowed? The timing of these two events looks like what is going wrong. This is perhaps something that could be discussed? It would also be nice to show a plot of the actual tracking data, the videos are a bit noisy. Do they see evidence for EB1 coming from the centrosomes towards the metaphase plate and comets going outwards from the metaphase plate? The point that might deserve discussion is whether Mms19 might be regulating microtubule nucleation from chromatin

or from existing microtubules or help stabilize this process?

➔ *Yes, it seems that MTs initially form over chromatin and that the centrosomal microtubule assembly is delayed. This can be seen in the supplementary movie S3 mov. At t=2min, we see a dense signal corresponding to MTs in an area which most likely corresponds to chromatin. This signal appears before any substantial MT growth can be seen from the centrosomes. We have now tracked individual particles in 10s projections and added these pictures to Fig 4, indicating for many of them the direction. We do see MTs going from centrosomes to the nucleus, and tips traveling from the metaphase plate towards centrosomes in WT and $Mms19^P$ NBs. In the discussion we have included our hypothesis of how Mms19 affects assembly of both centrosomal MTs and chromatin nucleated MTs during spindle assembly.*

Do Mms19 cells have apical microtubule asters in interphase, or is it purely mitotic as the CAK axis suggest, the authors must have these results in their data sets (live imaging)?

➔ *In most NBs that we analyzed, interphase asters can be seen in both WT and $Mms19^P$ NBs. Considering the apparent defects in Aurora A and TACC localization, we think that the astral MT assembly is more severely affected during entry into mitosis.*

Overall, however, the weakened spindles are convincing and the experiments to probe into this problem appear sound. Perhaps revise some wording in the figure legend of Fig 3. (e.g. line 949).

➔ *We have rephrased the wording as follows. To quantify spindle elaboration, spindle length was normalized to the cell diameter.*

Fig 4 C,D is a perhaps bit redundant with Fig 2, without quantification?

➔ *We removed this data and only kept the EB1 velocity graphs and images in Fig 4*

Fig 5:Line 287: Interestingly, whereas the Mms19::eGFP fusion protein was able to rescue this phenotype, CAK overexpression was unable to do so (Fig 5C-E).
I am not sure, but does the quantification in Fig 5E really show rescue? The statistical test is missing at least. From the micrographs I would think it looks pretty well rescued. Also, for CAK the test is missing to support that it does not rescue.

➔ *We decided against using the previous Fig 5E, because smaller cells always have smaller spindles. Instead, we now use only the spindle length relative to the cell diameter (Fig 3C) and we calculated the significance for all relevant pairs. We have included the statistical test and p-value for the graph comparing da>CAK vs $Mms19^P$ and Mms19::GFP vs $Mms19^P$ in the legend. For da>CAK, although we do see a partial rescue of spindle length, this is not statistically significant.*

Fig 7 . Mms19 binding to Tubulin interesting, but essential quantification is missing for microtubule bundling effect in the EM data, this could perhaps be improved.

➔ *We have now added a quantification for bundling in Fig 7I.*

Reviewer #2

Mms19 promotes spindle microtubule assembly in neural stem cells through two distinct pathways. By Chippalkathi, Egger and Suter.

In this study, the authors have characterized in vivo, using Drosophila, the phenotype of mms19 mutant during brain development. They have shown that the mms19 mutation causes microcephaly and impairs NB proliferation caused by a mitotic delay. Interestingly, mms19 cells exhibit mitotic spindle assembly defects and failure to

recruit the D-TACC/Msps complex at mitotic centrosomes. This particular defect can be rescued by overexpressing the CAK complex. The authors also describe that their mitotic phenotype, may to some extend also be caused by a direct effect of Mms19 protein on microtubule polymerisation or stabilisation, as suggested by in vitro experiments with pure tubulin and MTs.

In one hand, I think this is potentially interesting story. On the other hand, the way some of the experiments were done does not convincingly support the conclusions that are inferred.
In particular, the direct effect of Mms19 on MTs remains to be shown.

Major points:
1-Lane 183. There is confusion in the mitotic duration analysis. Mitotic timing is the time between NEBD and anaphase onset (which reflects the time to assemble a spindle and satisfy the spindle checkpoint). Cytokinesis completion is not appropriate to determine the mitotic timing, and cannot be determined by using EB1-GFP. I recommend analyzing all the movies to make new figures and calculate real mitotic duration (which is between 5 and 7 min in control NBs). Ideally, a double SAC+ mms19 (mad2) would shorten the mitotic timing and validate that the delay in M phase is caused by SAC activation.

➔We have now narrowed down the delayed period from measuring the length of the entire mitotic cycle to measuring the time between NEBD to anaphase B onset using a strategy that was verified in different publications (Cheerambathur et al, 2007; Lartigue et al, 2001; Wang et al, 2015). We were able to track the beginning of spindle elongation <u>and</u> the decrease of EB1::GFP intensity (happening synchronously at Anaphase B onset) from NBs progressing from metaphase to anaphase B. These measurements gave us qualitatively the same results as the previous ones. The "ideal" case scenario described by this reviewer was not possible to set up and perform in time for resubmission (and may also be difficult to perform over longer periods of time) because there are already many genetic elements involved in these experiments. Instead, we tested whether the SAC is still functional by investigating whether non-disjunction of the second chromosome increases in $Mms19^P$ neuroblasts. As we show in S1 Fig, lack of Mms19 activity does not significantly increase non-disjunction, strongly suggesting that the SAC is still active in these NBs. Considering also our other result on spindle assembly, this result and the close timely and causal linkage between the two anaphases (Parry and O'Farrell, 2001; Pereira and Schiebel, 2003; Woodbury and Morgan, 2007) strongly suggest that the mitotic delay is primarily caused by an impaired assembly of the fully functional bipolar spindle, keeping the SAC active for longer. (See also point 4 of reviewer 3 for an extended explanation).

2- I am not convinced by the in vitro studies presented here.
-Indeed, many proteins show the ability to bind tubulin in vitro (especially using such high concentration of proteins) that reflect unspecific interactions, aggregations. Can we see how "pure" is recombinant (His)6-Mms19 on a coomassie gel ? Is the prep contaminated by other proteins ?

➔ We now show this Coomassie Blue stained gel in Fig 7A. The amount of Mms19 protein is not high, but no other protein can be seen. We also would like to point out that this Mms19 protein was expressed in E. coli and that contaminations by E. coli proteins are less of a problem when working with MTs.

-Moreover the measured 340 nm OD for the tubulin polymerisation experiment rather suggests aggregation than polymerisation. Why tubulin doesn't not show spontaneous polymerisation on its own ? In a classical turbidity assay with 40 microM tubulin, the OD should reach 0.4.

➔ We have now removed this Figure and experiment, even though we are of the opinion that the control we had presented in the previous version showed that these were not aggregates, but polymers. MT polymers can be depolymerized by cooling them down, whereas aggregates would usually not dissolve at lower temperature. This control therefore seemed to be a good one. However, because it seems that we can convince the reviewers of our (now more cautiously phrased) conclusion, it seems that we can leave out this experiment in the new version.

-Interestingly the authors suggest that MTs are decorated by discrete particules in their EM pictures (Figure 7) supporting the hypothesis that Mssp19 would be a microtubule associated protein. These EM experiments frequently leads to artifacts (depending on protein purity).

➔As discussed above and already stated in the previous version of the manuscript, this Mms19 fraction was expressed in E. coli where one does not expect to co-purify MAPS. Furthermore, there is no other protein visible in the prep (aside from Mms19).

-The fact that Mms19 would be a MAP is not demonstrated in this manuscript :

-Interestingly, the authors do have a functional Mms19-GFP transgenic line. How is the protein localized in vivo ? Is it associated with MTs or spindles ? Their previous work published in Developmental Biology (2018), suggested it is a cytoplasmic protein. May be better pictures could be provided here with live NBs expressing Mms19-GFP in the mutant background.

➔Mms19 has several different functions and there is no doubt that it is (also) a cytoplasmic/cytosolic protein. The cytoplasmic localization is essential for its function in FeS delivery that has been documented well in the literature (and is summarized in our manuscript). Localizing Mms19 turned out to be difficult because of its levels, its presence in different compartments (reflecting at least in part its different functions), and the difficulty in getting specific antibodies (even the human Mms19 protein localization is controversial). Live imaging with fluorescently tagged Mms19 turned out to be difficult to interpret because Mms19::GFP signal was too weak, precluding clear determination of its localization. Images of fixed NBs expressing Mms19::eGFP, however, showed that the GFP signal was partially overlapping with the spindle MTs and we observed a slight enrichment on astral MTs (S5D Fig; indicated by arrows). Furthermore, we also determined the localization of Mms19::eGFP in differentiating neurons and found it to be heavily enriched in neurites, structures containing MT bundles. In these neurites, we also observe co-localization of Mms19:eGFP with MTs (S5E Fig).

- One would expect for a stabilizing protein/MAP that overexpression would lead to MT stabilization in vivo. Is it possible to overexpress Mms19 and analyze MT networks ?

➔We overexpressed Mms19 in NBs using a UAS-Mms19 construct driven by Insc-Gal4 but did not observe an obvious difference in spindle architecture. We can only speculate why this is, but we found also evidence that in vivo Mms19 activity might be regulated post-translationally. This we had mentioned in the discussion in the previous version, but have removed in this version as suggested by reviewers. (We agree that it was too speculative and distracting from the main points).

-It is indeed tempting to speculate, given the lower MT polymerisation speed of glial cells that Mmsp19 is involved in the control of MT dynamics but these brains are heavily affected by the loss of mms19 and it could be a secondary effect. The same remark can be made for the lack of neurites extensions in mms19-cultured neurons. To conclude, there are no convincing evidences that MMs19 regulates MTs on its own and is responsible for the second pathway regulating spindle assembly.

➔Removing the turbidity assay (which gave a very comparable result) from this version of the ms, unfortunately, weakens our argument and forces us to phrase it more cautiously. We have done this now. On the other hand - and as detailed above – it should be kept in mind that the experiments with the E. coli expressed and purified Mms19 showed direct binding to MTs. Furthermore, the E. coli expressed Mms19 also shows an effect on MT length, stability and bundling. We can accept that we need to phrase our conclusions a bit cautiously, but we feel that the evidence we present is stronger than judged by this reviewer.

3-Epistatic experiments show that in mms19 the main problem is a defective CDK1 activation and spindle assembly defects (likely because CAK remains sequestrated by XPD). This is not supported by figure S1 that reveals that mms19 mutant displays lower number of G2 cells and higher mitotic cells. We expect higher numbers in both categories.

➔This is an interesting and good point, and there could be several reasons for this. 1) This figure displays percentage of cells in the different phases (a raise in one group therefore needs to be compensated for by a drop in another group). 2) Cdk2 and Cdk4/6 also depend on CAK for their full activation (Mms19 downregulation would therefore allow fewer cells to enter and proceed through S.phase in Mms19P NBs). 3) Cells staining positively for both EdU and pH3 have passed S phase and could be in G2 or M. We cannot really discriminate between cells in G2 or M with this approach. This point convinced us that we should modify the display of these results and merge the two groups into one that we now called 'mitotic phase' (Fig S2D).

However, despite the absence of Mms19, cells manage to enter in mitosis suggesting Cdk1 can be still activated (therefore the model presented in figure S3 C is wrong, or at least too simple and should probably include other triggers of CDK1 activation).

➔Thank you for pointing this out. The model should not show an On/Off switch but should be more dynamic. We agree that there should be basal levels of Cdk1 activity, too, and we have now updated the model to show this (S6 Fig).

I feel that the mitotic phenotype phenotype seen here may be caused by a weaker cdk1 activation (as suggested by the fact that CAK overexpression rescues D-TACC recruitment). This could be challenged experimentally by FRET probes for CDK1 (but these are complicated and time experiments). Alternatively, immunostaining with phosphoantibodies for known CDK1 targets could be performed.

➔ We have actually shown previously (Nag et al, 2018) that phospho-Cdk1 levels are reduced in Mms19^{P} larval tissues. We now show the Aurora A localization in S4E,F Fig, instead. This also provides further support of our hypothesis that the Cdk1 activity is reduced in Mms19P.

I am also surprised that the lowering of XPD levels (an experiments that was presented in their previous study, Ma et al., 2018) is not shown here to fully challenge this hypothesis.

➔The problem with Xpd is that it is also required for transcription and the mutants die at L1. In the young embryo we could do this, because there is no transcription that is essential to reach cellularization. This is not the case for the larval brain. Finding the right dose and delivery for the NB experiment therefore seemed to be too difficult. Furthermore, the interpretation of the results of this difficult NB experiment would be trickier because in NBs we cannot rule out that a transcriptional effect (of reducing Xpd activity) causes the change in phenotypes.

I was also wondering if da>CAK induces spindle modifications and triggers brain development defects: this important control is lacking in all figures. It is possible that excess of CDK1 activity may shorten the spindle due to CAK overexpression since tissue growth seems sensitive to the GAL drivers used, at least in disks. It is therefore difficult to interpret the data.

➔We have now included this requested data in Figures 1, 3 and 5 where we overexpressed CAK using daughterless>Gal4 in the WT background and analyze the brain size, NB number and spindle assembly. These brains do not show elevated frequencies of defects compared to the WT.

Minor points that nevertheless need to be amended.

1-Please measure the angles between centrosomes and the center of the nuclei (just before NEBD) to quantify the centrosome separation failure (similarly to figure S4).

➔ *We have added this quantification in Fig2D,E.*

2-Result section: avoid information that should be in the material and method section (ex: lane 183-185, also lane 950).

➔*We have modified the text accordingly.*

4-In the graphs it is sometimes difficult to see which samples are compared in the statistical tests (the blue bar stops between 2 samples). It is also not clear to me if the central brain volume, the Number of NBs per lobes, the OL volume is different between mms19 and mmsp19, da>CAK. It appears different to me but the P value is not shown.

➔*We have now compared WT vs Mms19^{P}; Mms19^{P} vs da>CAK, Mms19^{P}; Mms19^{P} vs Mms19::eGFP, Mms19^{P} and WT vs da>CAK, WT. We also show p values in the legends (wherever possible) and have included a table showing the p values for all comparisons as supplementary data (S3 Table).*

5-This is a matter of taste but I feel the discussion is too long, does not go the point and distract the reader from the main message.

➔*We modified the discussion and have now put more emphasis on the contribution of Mms19 to the steps in spindle formation.*

Other points.

6-I would prefer to see dot plot (+/-sd) instead of histograms or box plots. The exact n for each sample analyzed should be included in the figure legends.

➔ *We changed the graphs to scatter plot-type, indicated SD and mentioned exact sample size in the legends*

7-The proteomic data are not needed. Why using a control that is also involved in MT dependent processes (lane 342)? Why not using GFP ? How the data can be interpreted ?

➔ *When we performed the MS we did not know about the possibility of direct spindle MT regulation by Mms19. Furthermore, we think it is a good service to the community to publish the proteomic data in the supplementary material, as, this was already appreciated by colleagues.*

8-I am not sure that the neurite experiment can be interpreted because the MT binding properties of Mms19 have not been demonstrated in this study. It could be a secondary effect.

➔ *We removed this data (and only left the pictures that show colocalization in neurites).*

9-How is Aurora A kinase localized in mms19 mutant cells ?

➔ *Aurora A localizes to centrosomes in WT NBs but is depleted from centrosomes in Mms19^{P} NBs (S4E,F Fig).*

10- Lane 440-443. I wouldn't say that their previous studies have clearly shown mms19 interaction with MTs (Nag et al., 2018).

➔*We removed this line*

Reviewer #3

The article by Chippalkatti and colleagues entitled: “Mms19 promotes spindle microtubule assembly in neural stem cells through two distinct pathways” investigates the role of Mms19 in neuroblast cell division in the Drosophila central brain. They initially show that Mms19 mutant neuroblasts generate fewer GMCs than WT neuroblasts. They show that Mms19 mutant neuroblasts assemble spindles that are less robust than WT spindles and spend more time in mitosis. They further show that Mms19 contributes to microtubule stability and possible to bundling. The identification of Mms19 as a contributor of accurate mitosis is interesting and novel. The specificity of the phenotype in neuroblasts is also quite interesting. However, somehow at the end of the study we do not understand several observations and the various observations appear disconnected in order to understand the findings described here. I think the authors should address some important points to make this study a stronger candidate for Plos Genetics. Otherwise, the article appears rather descriptive and preliminary.

Major points:

1- Why neuroblasts are more sensitive to the lack of Mms19? In other words, what makes these cells so sensitive to the loss of this MAP, when compared to other brain cells from the optic lobe, for instance?

➔This must be a misunderstanding. Neuroblasts simply turned out to be a well-suited system to study the role of Mms19 in a cell that cycles through a normal cell cycle. Mms19 regulates mitosis across different tissues and cell types. For example, we have previously reported severe mitotic defects in Drosophila young embryos and in imaginal discs (Nag et al, 2018). Although we did not analyze optic lobe neuroblasts or epithelial cells, we would predict to find similar effects because of the severe reduction of the size of the OL.

2- If neuroblast numbers are not that affected (Figure 1D), why the brain volume is decreased? Is the number of GMCs or neurons responsible for this? Also, when I look at the pictures shown in Figs 1A-B, it really seems that Mms19 mutant have fewer of these cells (Mira positive). The quantifications, however do not seem to show this. How do the authors explain this? I wonder if this relates with developmental stages. The extension of larval stages in the mutant might make a staged analysis more difficult, but maybe worth considering?

➔Due to slower mitotic divisions, the number of GMCs and neurons generated is lower and this probably contributes to the reduction in brain size. We have validated this by analyzing $Mms19^P$ MARCM clones that show fewer GMCs/neurons as compared to WT clones. The previous Fig 1A-B displayed the $Mms19^P$ brain where the Hoechst signal was saturated and it was therefore difficult to see the Mira positive NBs. We have now added a new picture for the $Mms19^P$ brain. We have tried to stage the larvae before, but the much slower development of the $Mms19^P$ genotype makes this difficult. Here we therefore focused on the mitotic divisions. Nevertheless, we have considered other reasons for the smaller brain and found this defect in neurite formation that we included in the previous version, but were encouraged to remove because it does not fit into the paper’s topic which focuses on mitosis. In other words, we think that there are other, developmental problems as well.

3- In Figure 2, I am not sure if understand what the authors mean by spindle orientation. Mis-orientation should be quantified towards a reference point, like a polarity crescent or the position of the daughter cells, which in this system should always be the same-see the Gonzalez lab papers.

➔We have analyzed the spindle angle relative to the basal crescent of the polarity protein Miranda. We have added this data in Figure 2 (F-J).

4- Why is mitosis prolonged in the Mms19 mutant neuroblasts? Is it dependent on the spindle assembly checkpoint? Since nevertheless these cells seem to exit mitosis, do they generate aneuploid neuroblasts? Can this be at the basis of the decrease in brain size?

➔All evidence we have points to Mms19 being required for the proper assembly and/or stability of spindle MTs. The observed decrease in MT velocity (Fig 4B,C) and MT density (Fig 3E-H), would cause centrosome migration and spindle orientation defects (Fig 2D-J), and delay the assembly of a normal spindle, thereby compromising kinetochore-MT

attachment and keeping the spindle assembly checkpoint (SAC) active. This would then delay progression into anaphase. Furthermore, in the Mms19^{P} NBs shown in S5B,C Fig. and in the suppl. movies S3 mov. and S8 mov., we see that MTs are assembled initially only from one centrosome, and only after 6-7 minutes post NEBD do we see an assembled bipolar spindle. In this situation, the SAC is predicted to be kept active even longer. We have performed fluorescent in situ hybridizations (FISH) to test for non-disjunction and thereby for SAC activity. We did not observe significantly elevated levels of aneuploidy (S1 Fig), suggesting that the SAC is still functional in these NBs.

5- I am not sure that I agree with the interpretations based on the data shown in Figure 5. The authors show spindles in that Mms19 mutant neuroblasts and controls revealed by a-tubulin labeling. The spindles appears indeed very different, smaller and less organized. But the authors state that that Mms19 is required for spindle repolymerization. To me it does not seem a problem of microtubule re-polymerization. The microtubule mass is quite impressive in the mutant situation, just not well sorted into a bipolar array. But if I look at time t-30s, the array emanating from the centrosomes appears quite impressive in the mutant and comparable to controls. Maybe the problem is in establishing the length and not in generating microtubules after depolymerization?

➔*We have now replaced re-polymerization by re-assembly and refer in a neutral way to the formation of a bipolar spindle after cold treatment. We certainly agree that there is good evidence for an activity promoting stability, length and bundling. Even though we observed an MT mesh at t=30s in the Mms19^{P} NBs, at t=90s, the spindle MT density appears still quite sparse as compared to WT density at t=90s. When we quantified spindle MT density in Mms19^{P} NBs not subjected to cold treatment (Fig 3H), we also observed a lower density. Furthermore, mov 08 and mov 03 show the delay in assembly of spindle MTs. Our view is therefore that the Mms19^{P} NBs in general contain fewer MTs and, as suggested by this reviewer, these MTs may not be properly organized. Spindles developing like this would take longer to push the centrosomes towards the cortex and to reach the length of WT spindles. We discuss this now in the discussion section.*

6- I wonder how the authors build a model around their data related with TACC. TACC, has a minor phenotype in neuroblasts. Could it be that there is some redundancy between the two proteins? Did they make double mutants? Along the same lines, the table of interactors does not show TACC or any major MAP with important roles in spindle assembly. However some motors are present, which might explain the "abnormal spindle phenotypes". Have they considered the option that maybe Mms19 plays a role in recruiting or activating a motor?

➔*This is an interesting question, we could not completely solve. We were able to show that Mms19 acts through CAK - Cdk1 – Arurora A – TACC/Msps, but that this is not its only activity because there seems to be also a CAK-independent activity. This CAK-independent function seems to act through the MT-binding activity of Mms19. However, we now reduced this part of the manuscript because we need to obtain stronger data in a postmitotic system to be able to clearly demonstrate this activity and this would then not fit into the mitotic story presented here. Interestingly, since our first submission, a paper was published that links Crumbs and Mip18/Galla (interacts with Mms19 in the cytoplasm for FeS delivery) with the fly kin5 homolog. Although we did not find this particular kinesin in our IP (however, the mitotic Mms19 complex might be a minor fraction in our wild type extract), it is still possible that Mms19 links to them and that this could be the TACC-independent activity of Mms19. We added this to the discussion.*

7- Several recent papers have analysed and compared mitotic spindles in different tissues or at different developmental stages. Maybe these should be included in either the introduction and or discussion?

➔ *We added a short review of this in the introduction and added the literature references.*

References:

Pereira, G., & Schiebel, E. (2003). Separase Regulates INCENP-Aurora B Anaphase Spindle Function Through Cdc14. *Science*. https://doi.org/10.1126/science.1091936

Parry, D. H., & O'Farrell, P. H. (2001). The schedule of destruction of three mitotic cyclins can dictate the timing of events during exit from mitosis. *Current Biology*. https://doi.org/10.1016/S0960-9822(01)00204-4

de Lartigue, J., Brust-Mascher, I., & Scholey, J. M. (2011). Anaphase B spindle dynamics in *Drosophila* S2 cells: Comparison with embryo spindles. *Cell Division*. https://doi.org/10.1186/1747-1028-6-8

Cheerambathur, D. K., Civelekoglu-Scholey, G., Brust-Mascher, I., Sommi, P., Mogilner, A., & Scholey, J. M. (2007). Quantitative analysis of an anaphase B switch: Predicted role for a microtubule catastrophe gradient. *Journal of Cell Biology*. https://doi.org/10.1083/jcb.200611113

Wang, H., Brust-Mascher, I., & Scholey, J. M. (2015). The microtubule cross-linker Feo controls the midzone stability, motor composition, and elongation of the anaphase B spindle in *Drosophila* embryos. *Molecular Biology of the Cell*. https://doi.org/10.1091/mbc.E14-12-1631

Hwang, J. H., Vuong, L. T., & Choi, K. W. (2020). Crumbs, Galla and Xpd are required for Kinesin-5 regulation in mitosis and organ growth in *Drosophila*. *Journal of Cell Science*. https://doi.org/10.1242/jcs.246801
