# Supplementary material for: Mms19 promotes spindle microtubule assembly in Drosophila neural stem cells
Source: PLoS Genet. 2020 Nov 19;16(11):e1008913. doi: 10.1371/journal.pgen.1008913 (PMC7714366; doi:10.1371/journal.pgen.1008913)
Supplement: Attachment — Submitted filename: Comments to the Authors and responses.pdf [file pgen.1008913.s019.pdf]

Comments to the Authors:

Please note here if the review is uploaded as an attachment.

Reviewer #1: This is a well executed study with interesting results that are of relevance to the readership of Plos Genetics.

The manuscript has now greatly improved, but I feel the authors should clarify a few things before publication.

FIg1: Line: 182: "These observations indicated that the Mms19P
183 CB NBs probably did not proliferate enough to produce the normal amount of neuronal tissue [...]."

I still think that Fig 1 still shows that the CB is not so much affected, but that mitosis in the neuroepithelium is strongly reduced (PH3 signal/ Fig 1B the red area changes much less than the green). When you do live imaging on brains , it is obvious that the neuroepithelium and the neuroblasts it produces divide a lot. The authors should say that the brain size defects are more likely to stem from defects in highly proliferative areas like the neuroepithelium. If they want to insist that the slower CB neuroblast divisions are causing this, they need to do more experiments using Gal4 drivers that allow specifically testing CB neuroblast rescue in Mms19p mutants. It is a less elegant link to the subsequent experiments on neuroblasts. But those are still very justifiable and a very good system to understand mechanics that regulate spindle assembly.

>>Thank you for pointing this out. This is indeed quite evident. We have now rephrased this part (line 161-190; 205-208 in the new manuscript).

Fig 2: I now realise that Fig 2G right panel is quite unlucky, is this the best picture showing spindle misalignment? I am to so sure it does. If they had a better one that would be desirable. As their interpretation that the degree of spindle misalignment is not in the range to cause neuroblast amplification (which is rather due to inheriting aPKC than Miranda symmetrically (Doe lab), perhaps rephrase that?). The interpretation/ conclusions here is nonetheless valid, i.e. spindle orientation defects are unlikely to explain it.

>>We have now changed the picture for *Mms19*^{P} NB in Fig2G and have rephrased the connection between the spindle positioning and cell fate determination/differentiation, putting the emphasized properly onto the role of aPKC in retention of NB identity (line 271-273; 281-283).

Fig 5:
line 331: "With this procedure, the NB spindles were completely depolymerized after incubation on ice for 30 min (Fig 5A)". Either add picture, or remove (Fig 5A) and say "not shown".

>>Here we refer to the leftmost panel in Fig 5A which shows a WT NB where we visualized MTs immediately after 30min cold incubation i.e. 0 sec. We have now mentioned “left panel”, “center panel”, and “right panel” to refer to MTs visualized after 0s, 30s, and 90s incubation at 25°C, respectively. We also removed the word “completely” from the text because very short MT extensions are visible.

Reviewer #2: In this revised version, the authors have answered my questions and the new manuscript is much more focused on the important points. Moreover, clear efforts have been made on the figures.

I have a last minor points, (which will not require me to review a last this manuscript): It might be desirable to indicate that Aurora A also controls the degradation of cyclic B and

add the corresponding reference (Caous et al., 2015), which may explain in part the increase in mitosis time.
>>Thanks for the suggestion. It is an interesting point and we have now added this connection in the discussion.

Aside from these changes we noticed and corrected a minor error in the numbers used in Fig 1C and a couple of typos.
